# Methylation of the Vitamin D Receptor (VDR) Gene, Together with Genetic Variation, Race, and Environment Influence the Signaling Efficacy of the Toll-Like Receptor 2/1-VDR Pathway

**DOI:** 10.3389/fimmu.2017.01048

**Published:** 2017-09-11

**Authors:** Vanessa Meyer, Donovan Sean Saccone, Fidele Tugizimana, Furaha Florence Asani, Tamsyn Jacki Jeffery, Liza Bornman

**Affiliations:** ^1^Department of Biochemistry, University of Johannesburg, Johannesburg, South Africa

**Keywords:** VDR, DNA methylation, TLR2/1, Vitamin D, polymorphism, cathelicidin, race, UVI

## Abstract

**Background:**

The disparity in prevalence of infectious diseases across the globe is common knowledge. Vitamin D receptor (VDR)-mediated toll-like receptor (TLR) 2/1 signaling produces antimicrobial peptides, which is critical as a first line of defense in innate immunity. Numerous studies disclosed the independent role of genetic polymorphisms in this pathway, vitamin D status or season and more recently epigenetics, as factors contributing to infectious disease predisposition. Few studies address the interaction between environment, genetics, and epigenetics. Here, we hypothesized that VDR-mediated TLR2/1 signaling is influenced by a combination of environment, epigenetics and genetics, collectively influencing differential innate immunity.

**Methods:**

Healthy Black and White South Africans (*n* = 100) donated blood, while ultraviolet index (UVI) was recorded for the duration of the study. LC-MS/MS supported 25(OH)D_3_ quantification. Monocyte/macrophage cultures, supplemented with/without 1,25(OH)_2_D_3_, were activated with the TLR2/1 elicitor, Pam_3_CSK_4_. *VDR*, cathelicidin antimicrobial peptide, hCAP-18, and 25-hydroxyvitamin D_3_-24-hydroxylase expression were quantified by RT-qPCR or flow cytometry. Pyrosequencing facilitated *VDR* methylation analysis and single-nucleotide polymorphism (SNP) genotyping in regions pinpointed through a bioinformatics workflow.

**Results:**

Season interacted with race showing 25(OH)D_3_ deficiency in Blacks. UVI correlated with 25(OH)D_3_ and *VDR* methylation, likely influencing race differences in the latter. Regarding the TLR2/1 pathway, race differences in SNP genotype distribution were confirmed and functional analysis of VDR-mediated signaling showed interaction between race, season, and 25(OH)D_3_ status. Multivariate OPLS-DA mirrored several interactions between UVI, 25(OH)D_3_ status, DNA sequence, and methylation variants. Methylation of the third cytosine-phosphate-guanine dinucleotide (CpG) in the promoter CpG island (CGI) 1062, CGI 1062 CpG 3, significantly discriminated a 5.7-fold above average mean in VDR protein level upon TLR2/1 elicitation, the variation of which was further influenced by 25(OH)D_3_ status and the *VDR* SNP *Taq*I.

**Conclusion:**

Regulation of VDR-mediated TLR2/1 signaling is multifactorial, involving interaction between environment [UVI and consequent 25(OH)D_3_ status], epigenetics (*VDR* methylation at key regulatory sites), and genetics (*TLR1, TIRAP*, and *VDR* SNPs).

## Introduction

In addition to its role in maintaining calcium–phosphorus homeostasis, vitamin D is a potent modulator of both innate and adaptive immunity, is involved in the regulation of cell growth and differentiation, detoxification of xenobiotics, and activation of monocytes/macrophages (1, 2). These actions of vitamin D are almost entirely dependent on the interaction between the most biologically active form of vitamin D, 1,25-dihydroxyvitamin D_3_ [1,25(OH)_2_D_3_], and the vitamin D receptor (VDR) transcription factor. Bound to 1,25(OH)_2_D_3_, the VDR regulates the expression of a myriad of genes (3, 4). Cathelicidin antimicrobial peptide (*CAMP*) and 25-hydroxyvitamin D_3_-24-hydroxylase (*CYP24A1*) are examples of two well-characterized vitamin D target genes, respectively, encoding the cathelicidin antimicrobial peptide (hCAP-18) and multifunctional vitamin D catabolizing enzyme.

Toll-like receptor 2/1 (TLR2/1) triggering activates a signaling cascade inducing both *VDR* (5) and *CYP27B1* in monocytes/macrophages. CYP27B1 catalyzes *de novo* production of 1,25(OH)_2_D_3_ from accumulated 25(OH)D_3_; delivered to the cells *via* the vitamin D binding protein (DBP), encoded by *GC*. The liganded VDR–transcription factor complex binds to vitamin D response elements (VDREs) in *CAMP*, activating *CAMP* expression and the production of hCAP-18. hCAP-18 is synthesized as a proprotein consisting of an N-terminal cathelin domain and a C-terminal LL-37 domain (6). While the cathelin domain is a cysteine protease inhibitor with broad spectrum antibacterial activity (7), LL-37 directly inhibits mycobacterial replication (5, 8), has antifungal activity against *Candida albicans* (9), and antiviral activity against HIV (10).

Since *CAMP* expression is dependent on vitamin D, and vitamin D deficiency has been linked to several infectious diseases including tuberculosis (11), sepsis (12), bacterial infections after kidney transplants (13), and HIV (14), it is not surprising that more than 100 clinical trials have assessed the efficacy of vitamin D supplementation as adjunct therapy in the treatment of various infectious diseases. However, the outcome of clinical trials has been conflicting and this is often attributed to differences in study design, baseline vitamin D status of participants, and outcome measurements. In fact, it appears that individuals can be classified into three groups: (i) those with a low response, (ii) those with a medium response, and (iii) those with a high response to vitamin D supplementation (15). These interindividual differences may result from variation in the regulation of *VDR* expression at both a genetic and epigenetic level (16). For example, VDR function to transactivate *CAMP* is influenced by the VDR single nucleotide polymorphism (SNP) *Fok*I (rs2228570) and ethnicity (17), while vitamin D insensitivity in breast cancer cells has been attributed to CpG methylation of the *VDR* primary promoter (18). Thus, both genetics and epigenetics have the potential to influence the response to vitamin D. Indeed, a double-blind randomized controlled trial assessing the impact of high-dose vitamin D_3_ during intensive-phase antimicrobial treatment of pulmonary tuberculosis showed that vitamin D only increased the time for sputum culture conversion in participants carrying the CC genotype of the *VDR* SNP *Taq*I (rs731236) (19). Additionally, *VDR* expression is influenced by the environment. For example, narrow-band UVB induces miRNA-125b (20), which directly regulates *VDR* mRNA translation, decreasing VDR protein level (21, 22). Seasonal variation in ultraviolet index (UVI) further correlates with circulating vitamin D (23). Thus, seasonal variation in UVI directly influences VDR function by altering the availability of the free 1,25(OH)_2_D_3_. Indirectly, changes in 1,25(OH)_2_D_3_ concentration may itself regulate *VDR* expression through multifunctional, 1,25(OH)_2_D_3_-responsive, enhancers located within the *VDR* itself (24). The complex regulation of the *VDR* through genetics, epigenetics, and environment (16) may therefore provide insight into inter-individual variation in response to vitamin D and the efficacy of vitamin D to enhance immune function.

Here, we evaluate (1) the effect of *VDR* methylation on the TLR2/1-VDR signaling pathway and (2) the impact of genetic and environmental factors on differential immune signaling. It was hypothesized that VDR-mediated TLR2/1 signaling is influenced by a combination of environment, epigenetics and genetics, collectively influencing differential innate immunity in healthy South Africans. Using an *in vitro* model, stimulating monocytes from healthy individuals with a TLR2/1 elicitor, we avoided pathogen-mediated changes in DNA methylation (25, 26).

Results presented here provide support for multifactorial regulation of VDR-mediated, TLR2/1 signaling, involving interaction between environment, epigenetics, and genetics. UVI influences 25(OH)D_3_ status, which regulates VDR expression through *VDR* methylation, while enhancing the extent and rate of VDR transactivation of *CAMP* encoding the antimicrobial peptide hCAP-18.

## Materials and Methods

### Sample Collection and Environment

In accordance with the Declaration of Helsinki, the Human Research Ethics Committee of the South African National Blood Service (SANBS HREC clearance certificate number 2010/01) and the Ethics Committee, Faculty of Science, University of Johannesburg (2010/06/03) approved the study. After written informed consent, the SANBS collected blood from randomly selected healthy Black (*n* = 50; age 17–62 years; 25 males and 25 females) and White (*n* = 50; age 17–69 years; 25 males and 25 females) South Africans living in Gauteng, SA. Samples were collected across all seasons, though no White individuals were collected in winter for functional analysis. UVI was obtained from the South African Weather Service weather station in Irene, Pretoria, Gauteng. As 25(OH)D_3_ has a half-life of 2–4 weeks in circulation (27), the approximate UVI that each individual could have been exposed to was calculated as the 4-week average before blood collection, using the average hourly UVI between 11.00 a.m. and 14.00 p.m. across the years of sample collection (2011–2014).

### Quantification of Circulating 25(OH)D_3_

Liquid chromatography tandem mass spectrometry (LC-MS/MS) facilitated quantification of 25(OH)D_3_ concentration in the Department of Clinical Biochemistry, University Hospital of South Manchester (UK), including four human serum pools from the Vitamin D External Quality Assessment Scheme (DEQAS, UK). A concentration of ≥50 nmol/L was considered normal/sufficient (28). 25(OH)D_3_ concentration was below the detection limit (3 nmol/L) in four Black samples, while sample was insufficient for six Blacks and eight Whites.

### Bioinformatics

To identify putative functional loci that could influence *VDR* expression and function through genetic and/or epigenetic mechanisms, a bioinformatics workflow was developed (Methods S1.1, Figures S1 and S2, and Table S1 in Supplementary Material).

### DNA Methylation Analysis

Monocytes were isolated and gDNA extracted, at time zero, as previously described (17). *VDR* methylation analysis by bisulfite pyrosequencing was outsourced to EpigenDx, Inc. (MA, USA). Selected sites typed included 10 CpGs in CpG island (CGI) 1066 spanning enhancer U3 (chr12:48340628-48340806, hg19), 56 CpGs in CGI 1062 spanning the primary promoter (chr12:48299359-48298799), 12 CpGs in CGI 1061 spanning exon 3 (chr12:48258845-48259024), and 18 CpGs in CGI 1060 spanning exon 9 (chr12:48238512-48238799). CpGs were numbered in the 5′–3′ direction.

### Genotyping

Genotyping by pyrosequencing was outsourced to EpigenDx, Inc. (MA, USA). Typed SNPs included *GC* rs7041, rs4588, and rs146681395; *TLR1* rs5743551 (A7202G), rs146940675, rs4833095 (N248S), rs111807776, rs143576765, rs5743618 (I602S), rs151036585, rs5743613 (P315L), rs185747096, rs146782074, and rs200631178; *TLR2* rs3804099 (T597C); *TiRAP* rs8177374 (S180L) and rs141792148; *VDR* rs11168312, rs11568820 (Cdx-2), rs182743714, rs184448883, rs4516035 (GATA), rs2228570 (*Fok*I), rs187018098, rs71951818, rs1544410 (*Bsm*I), rs7975232 (*Apa*I), rs731236 (*Taq*I), and rs4987032; *CYP24A1* rs6068812; and *DMNT3A* rs1550117 and rs112621472.

### Monocyte/Macrophage Culture and Treatment

To estimate TLR2/1 pathway efficacy, *VDR* mRNA, VDR protein, *CAMP* mRNA, hCAP-18 peptide, and *CYP24A1* mRNA, hereafter referred to as functional variables, were quantified following different treatments of monocyte/macrophage cultures that were established as previously described (17). Some monocytes were retained for functional analysis at time zero (baseline). The rest were settled in culture for 16 h prior to 24 h treatment with the vehicle control, 1,25(OH)_2_D_3_ (10 nM, Sigma Aldrich, St Louis, MO, USA), the TLR2/1 elicitor Pam_3_CSK_4_, (6.5 µg/ml culture media, EMC microcollections, Tuebingen, Germany), or both the elicitor and 1,25(OH)_2_D_3_.

### mRNA and Protein Quantification

The relative level of *VDR, CAMP*, and *CYP24A1* mRNA was quantified by RT-qPCR and VDR protein and intracellular hCAP-18 peptide by flow cytometry as previously described (17). Gene normalization was performed against two stably expressed reference genes: ubiquitin C (*UBC*) and tyrosine-3-monooxygenase/tryptophan-5-monooxygenase activation protein, zeta polypeptide (*YWHAZ*). Gene expression was quantified using the comparative CT method according to the MIQE guidelines, using inter-run calibrators and qBASE^PLUS^ software. To compensate for variability in fluorescence readings between experiments on the flow cytometer, the median fluorescence intensity (MFI) of broad-spectrum calibration beads was used to normalize data and thereby provide a calibrator for instrument-related variation in the flow cytometry readings over time. For hCAP-18, mouse IgG_1_ anti-human hCAP-18 primary antibodies (10 µg/ml, Abcam, Cambridge, UK) and APC-conjugated goat anti-mouse IgG1 secondary antibodies (2 µg/mL, Abcam, Cambridge, UK) were used. Western blotting facilitated tracing of hCAP-18 processing and secretion (Methods S1.2 in Supplementary Material).

### Statistical Analysis

IBM^®^ SPSS^®^ Statistics (v 23; SPSS Inc., Chicago, IL, USA) and SIMCA (v 14; Umetrics, Umea, Sweden) facilitated statistical analysis. Normal distributions were obtained by natural logarithm (ln) transformation of all functional data, except VDR protein. Correlation coefficients were computed using Pearson or Spearman’s rho. A general linear model was used for multivariate analysis of variance to assess main effects and factor interaction. Mann–Whitney *U* tested methylation differences. Pearson’s Chi-square test for independence assessed SNP distribution. Orthogonal projections to latent structures discriminant analysis (OPLS-DA, Methods S1.3 and Figures S3 and S4 in Supplementary Material) facilitated the study of the multivariate effect of *VDR* methylation, SNPs, and environment on TLR2/1-VDR signaling.

## Results

### Differences in Plasma 25(OH)D_3_ Concentration Are Influenced by Race and Season

**Figure 1:**
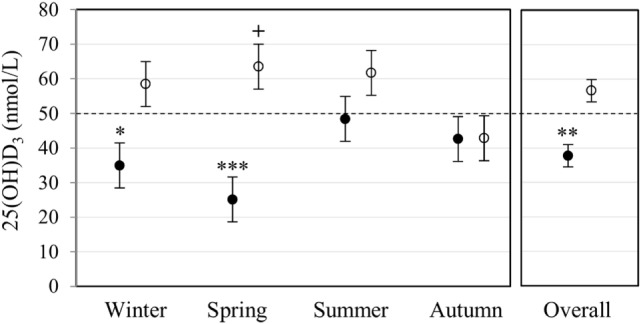
Race and season influenced plasma 25(OH)D_3_ concentration. The error bar plot shows the mean plasma 25(OH)D_3_ concentration, quantified by liquid chromatography tandem mass spectrometry, for healthy Black (filled markers, *n* = 50) and White (open markers, *n* = 53) South Africans, collected in winter (Black *n* = 17, White *n* = 10), spring (Black *n* = 10, White *n* = 16), summer (Black *n* = 10, White *n* = 11), and autumn (Black *n* = 13, White *n* = 16). Blacks were deficient (<50 nmol/L), having a significantly lower plasma 25(OH)D_3_ concentration than Whites in winter (*P < 0.050), spring (***P < 0.001), and overall (**P < 0.010). Overall, whites had normal 25(OH)D_3_ levels (≥50 nmol/L, ≤125 nmol/L), with a significantly higher plasma 25(OH)D_3_ concentration in spring compared to autumn (+*P* < 0.050). *P*-Values were adjusted using Bonferroni correction. Error bars show the unadjusted least significant difference at *P* < 0.050. The dotted line indicates the border between normal and deficient.

Plasma 25(OH)D_3_ concentration was quantified by LC-MS/MS (Figure 1). Race had a significant main effect on 25(OH)D_3_ concentration (*P* < 0.001). Overall, Blacks were deficient (<50 nmol/L) with a significantly lower 25(OH)D_3_ concentration (*P* < 0.010) than Whites, who had normal levels (≥50 nmol/L). Race interacted significantly with season (*P* < 0.010), showing a lower 25(OH)D_3_ concentration in Blacks in winter (*P* < 0.050) and spring (*P* < 0.001), but not in summer and autumn.

### *VDR* Methylation Differs between Races

**Figure 2:**
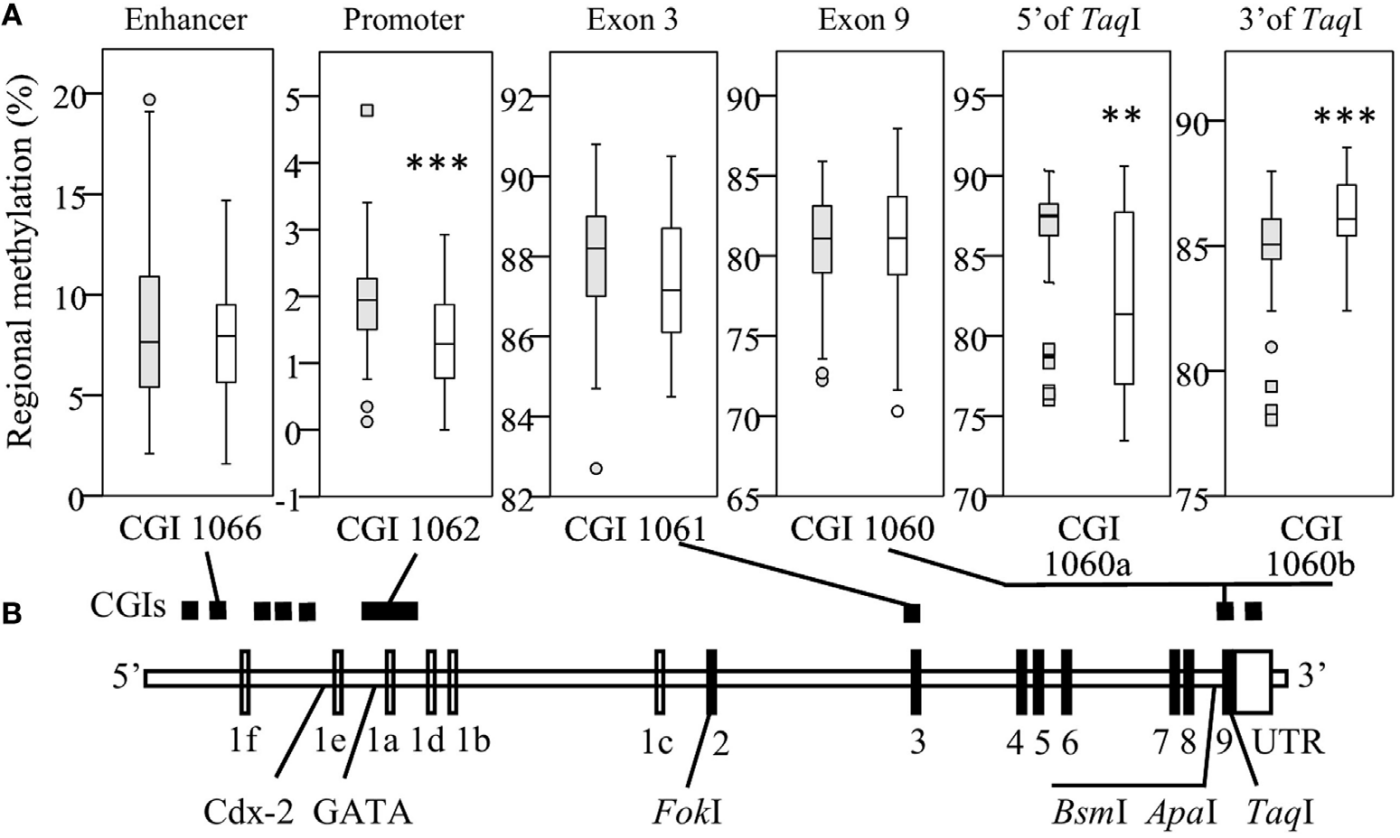
Race differences in regional vitamin D receptor (*VDR*) methylation. Box-and-whisker plots show the spread of regional methylation, quantified by bisulfite pyrosequencing, at the selected CpG islands (CGIs) **(A)** in context of *VDR* non-coding (unfilled) and coding exons (filled) and key single-nucleotide polymorphisms **(B)**; CGI 1066 spanning an enhancer, 1062 the primary promoter, 1061 exon 3 and 1060 exon 9, and the promoter of a non-coding transcript. Compared to Black South Africans (filled boxes, *n* = 50), methylation in Whites (open boxes, *n* = 50) was significantly lower in CGI 1062 (***P < 0.001) and 1060a (**P < 0.010), but higher in CGI 1060b (***P < 0.001). Box-and-whisker plots show the median (line in box), interquartile range (IQR, height of box), 95% CI (whiskers, extending to 1.5 IQRs or minimum and maximum values if no case has a value in that range), outliers (○, between 1.5 IQRs and 3 IQRs from the end of a box), and extreme outliers (◻, more than 3 IQRs from the end of a box). Site-specific methylation is shown in Figures S5A–D in Supplementary Material.

To assess the impact of *VDR* methylation on TLR2/1-VDR signaling, *VDR* methylation was quantified by bisulfite pyrosequencing. Regional methylation (Figure 2A) was compared between Blacks and Whites across the *VDR* in key CGIs identified through a bioinformatics workflow (Methods S1.1, Figures S1 and S2, and Table S1 in Supplementary Material). Comparing regional methylation, Whites had significantly lower levels at CGI 1062 (*P* < 0.001) and CGI 1060a (CpG 1-5, 5′ of *Taq*I, Figure 2A, *P* < 0.010) than Blacks, but higher levels at CGI 1060b (CpG 7-18, 3′ of *Taq*I, Figure 2A, *P* < 0.001). Significant racial differences in site-specific methylation (Results S2.2 and Figure S5 in Supplementary Material) were common in CGI 1062 (27/56) and 1060 (10/18), but less so in CGI 1066 (1/10) and1061 (3/12).

### TLR-VDR Pathway Genetics Differs between Races

To assess genetic variation between individuals in the TLR2/1-VDR signaling pathway, SNPs in several genes of the pathway and in the *de novo* methyltransferase enzyme, *DNMT3A*, were genotyped by pyrosequencing. Several SNPs, including *DNMT3A* SNPs, were monomorphic in the study population. Except for *VDR Bsm*I and *Taq*I, the frequency distribution of polymorphic SNPs differed significantly between Blacks and Whites (Table 1). The 1000 Genomes Deep Catalog of Human Genetic Variation confirmed race-specific genotype frequency distribution, also for *Bsm*I and *Taq*I though not for *Apa*I, comparing Africans (Yoruba) and Caucasians (Table S2 in Supplementary Material).

**Table 1:** Differential genotype distribution for polymorphic single-nucleotide polymorphism (SNPs) in the toll-like receptor (TLR)–vitamin D receptor (VDR) pathway in Black and White South Africans.

Gene	Genotype^a^	Black	White	Pearson’s chi-square			
			
Polymorphism (common name)		Number (%)	Number (%)	χ^2^	*df*	*P*-value	Cramer’s *V*^b^
***GC***							
rs7041	GG	1 (2)	10 (22)	60	2	<0.001	0.806
	GT	2 (4)	30 (65)				
	TT	43 (93)	6 (13)				
rs4588	AA	2 (4)	1 (2)	8	2	<0.050	0.298
	AC	6 (13)	18 (39)				
	CC	38 (83)	27 (59)				

***TLR1***							
rs5743551 (A7202G)	AA	1 (2)	26 (53)	61	2	<0.001	0.790
	AG	6 (12)	19 (39)				
	GG	43 (86)	4 (8)				
rs4833095 (N248S)	AA	2 (4)	24 (52)	43	2	<0.001	0.684
	AG	9 (20)	19 (37)				
	GG	34 (76)	5 (11)				
rs5743618 (I602S)	CC	0 (0)	0 (0)	57	1	<0.001	0.796
	CA	2 (4)	40 (83)				
	AA	45 (96)	8 (17)				

***TLR2***							
rs3804099 (T597C)	CC	28 (64)	12 (25)	21	2	<0.001	0.484
	CT	15 (34)	22 (47)				
	TT	1 (2)	13 (28)				

***TIRAP***							
rs8177374 (S180L)	AA	0 (0)	1 (2)	16	2	<0.001	0.402
	AG	1 (2)	15 (31)				
	GG	47 (98)	33 (67)				

***VDR***							
rs11568820 (Cdx-2)	AA	25 (57)	3 (6)	43	2	<0.001	0.683
	AG	17 (39)	14 (29)				
	GG	2 (5)	31 (65)				
rs4516035 (GATA)	AA	44 (90)	16 (33)	34	2	<0.001	0.587
	AG	4 (8)	28 (57)				
	GG	1 (2)	5 (10)				
rs2228570 (*Fok*I^c^)	TT/ff	2 (4)	12 (24)	17	2	<0.001	0.413
	TC/Ff	12 (25)	21 (43)				
	CC/FF	35 (71)	16 (33)				
rs1544410 (*Bsm*I)	AA/BB	2 (4)	8 (16)	4	2	ns	0.213
	AG/bB	21 (43)	20 (42)				
	GG/bb	26 (53)	20 (42)				
rs7975232 (*Apa*I)	TT/AA	27 (55)	13 (27)	12	2	<0.010	0.351
	TG/Aa	21 (43)	27 (55)				
	GG/aa	1 (2)	9 (18)				
rs731236 (*Taq*I)	TT/TT	21 (43)	21 (43)	2	2	ns	0.152
	TC/Tt	23 (47)	18 (37)				
	CC/tt	5 (10)	10 (20)				

*^a^Alleles used in genotypes represent those of the reverse strand on which the gene is located*.

*^b^Cramer’s V indicates effect size and varies between 0 and 1: small 0.07–0.20, medium 0.21–0.34, and large 0.35–1.00*.

*^c^*Fok*I, *Bsm*I, *Apa*I, and *Taq*I genotypes show nucleotides/restriction sites presence (lowercase) or absence (capital)*.

### Multivariate Effects of Race, Plasma 25(OH)D_3_ Status, Season, and Treatment on Functional Variables upon Elicitation of TLR2/1-VDR Signaling

**Figure 3:**
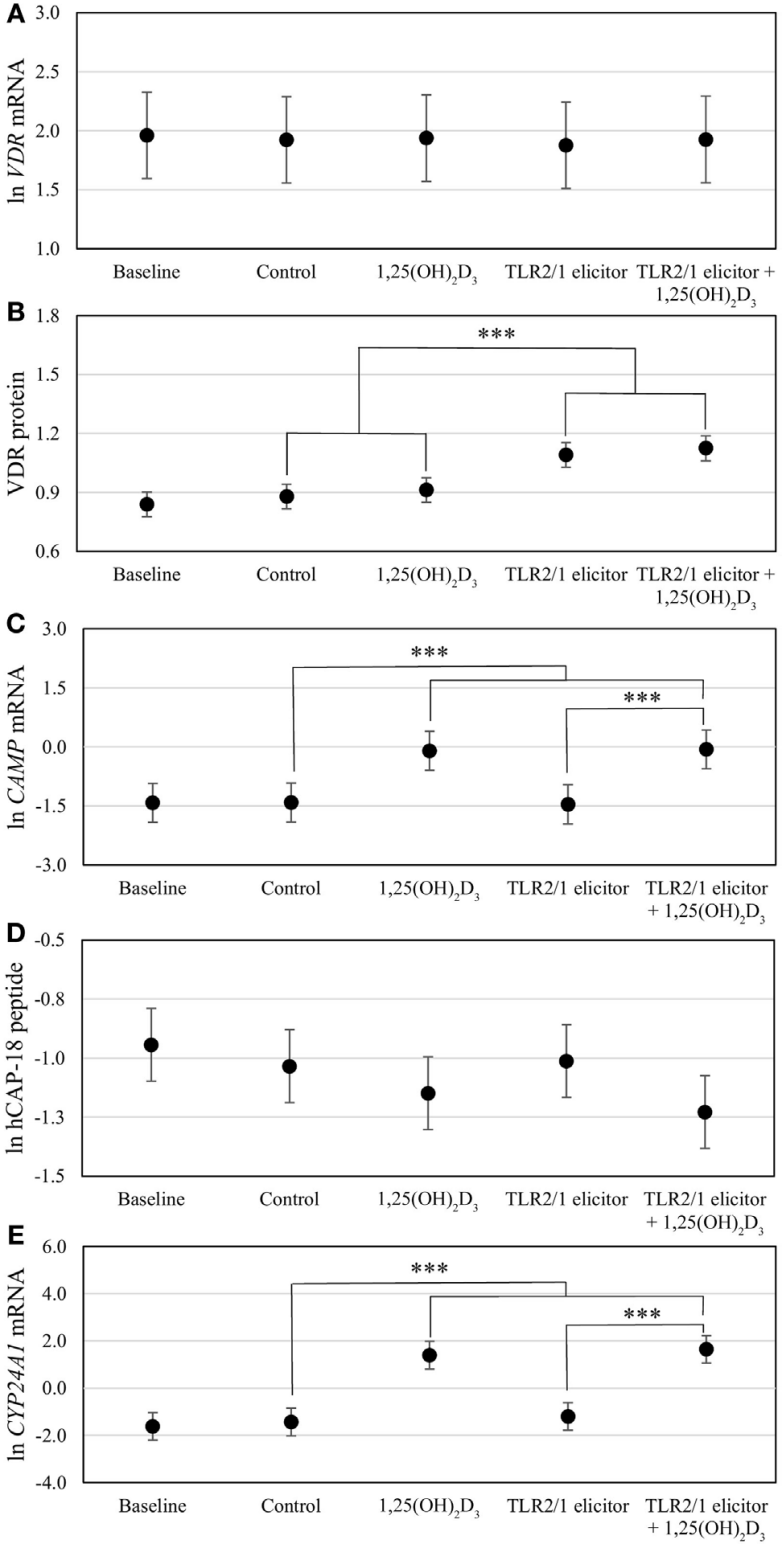
Differential vitamin D receptor (VDR)-mediated innate immune response to toll-like receptor (TLR) 2/1 elicitation, with or without *in vitro* 1,25(OH)_2_D_3_ supplementation. The error bar plots show mean levels for *VDR* mRNA **(A)**, VDR protein **(B)**, *CAMP* mRNA **(C)**, hCAP-18 peptide **(D)**, and *CYP24A1* mRNA **(E)**, for healthy South Africans (*n* = 100). Gene expression (mRNA) was quantified by RT-qPCR and protein or peptide level by flow cytometry. Significant treatment effects are shown (***P < 0.001). All significant differences were maintained after Bonferroni correction. Error bars show the least significant difference at *P* < 0.050.

**Figure 4:**
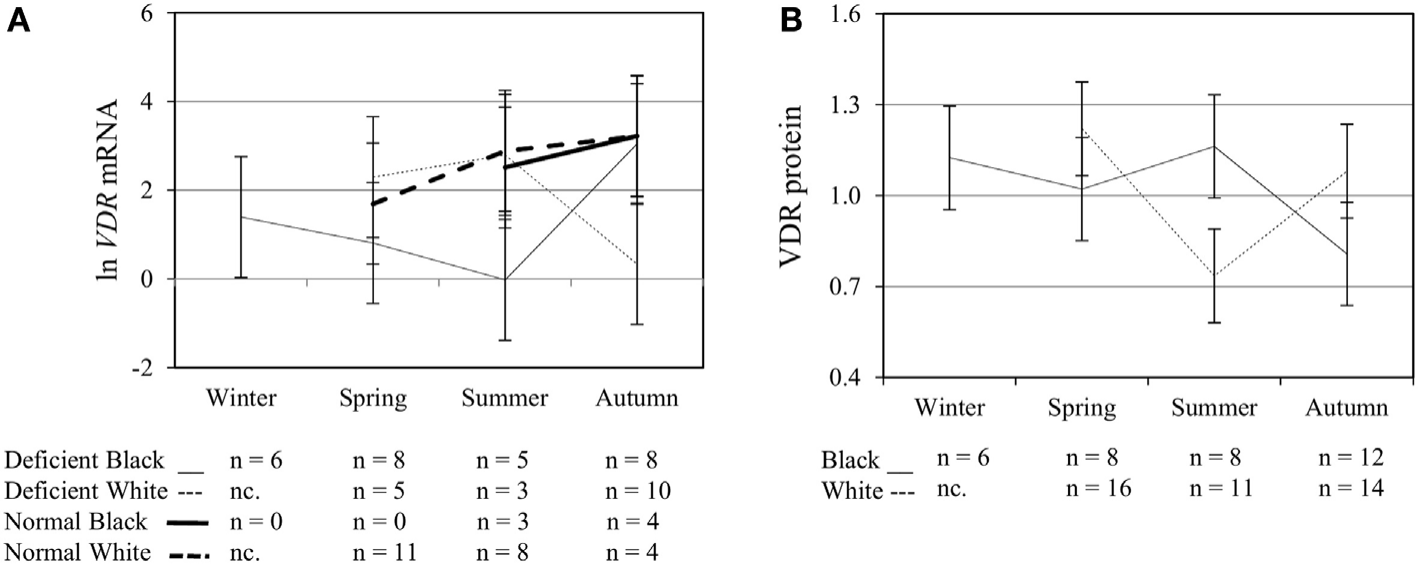
Interaction between season, race, and 25(OH)D_3_ status influence vitamin D receptor (*VDR*) mRNA and protein level. The line graphs show the interaction effects for *VDR* mRNA **(A)** and protein **(B)** level, quantified by RT-qPCR and flow cytometry, in monocytes/macrophages from healthy Black (*n* = 34) and White (*n* = 41) South Africans. Season, race, and 25(OH)D_3_ status interacted significantly to influence *VDR* mRNA (*P* < 0.001), while season and race showed interaction effects on VDR protein (*P* < 0.001). Error bars show the least significant difference at *P* < 0.050. nc. = not collected.

**Figure 5:**
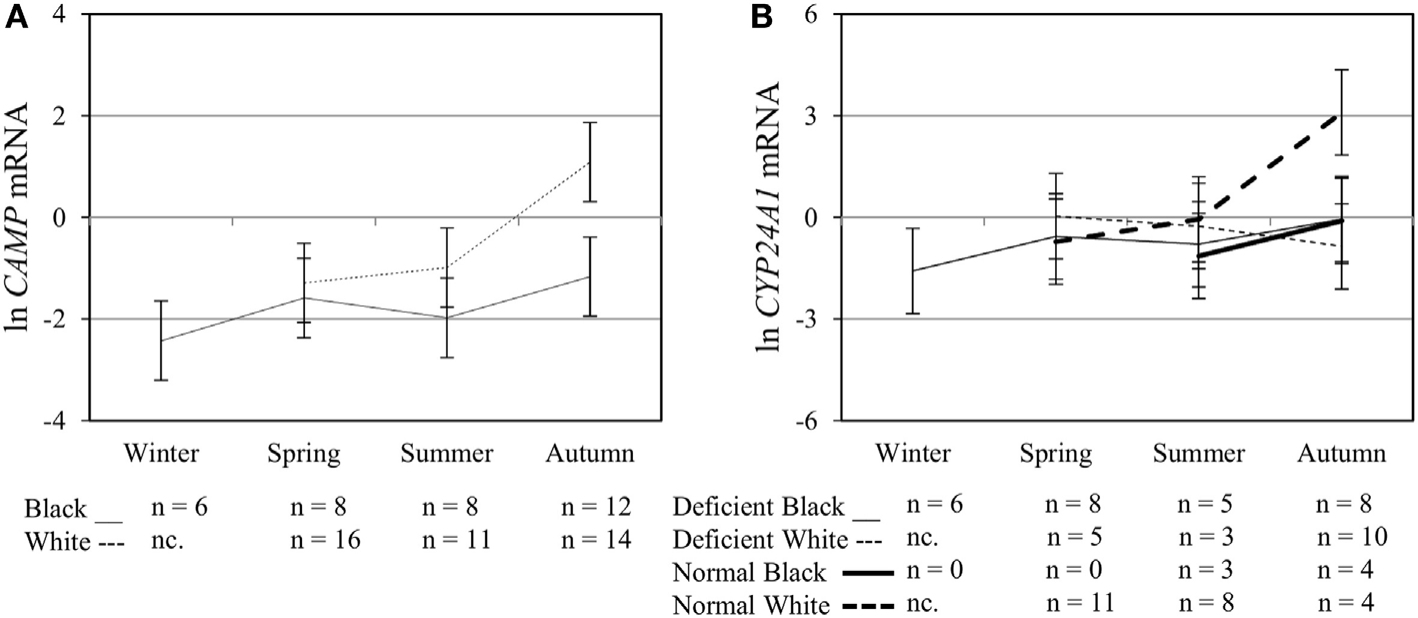
Interaction between season, race, and 25(OH)D_3_ status influence vitamin D receptor (VDR) transactivation of cathelicidin antimicrobial peptide (*CAMP*) and 25-hydroxyvitamin D_3_-24-hydroxylase (*CYP24A1*). The line graphs show the interaction effects for *CAMP* mRNA **(A)** and *CYP24A1* mRNA **(B)** level, quantified by RT-qPCR, in monocytes/macrophages from healthy Black (*n* = 34) and White (*n* = 41) South Africans. Season and race interacted significantly to influence *CAMP* mRNA (*P* < 0.050), while season, race, and 25(OH)D_3_ status showed interaction effects on *CYP24A1* mRNA levels (*P* < 0.050). Error bars show the least significant difference at *P* < 0.050. nc. = not collected.

**Figure 6:**
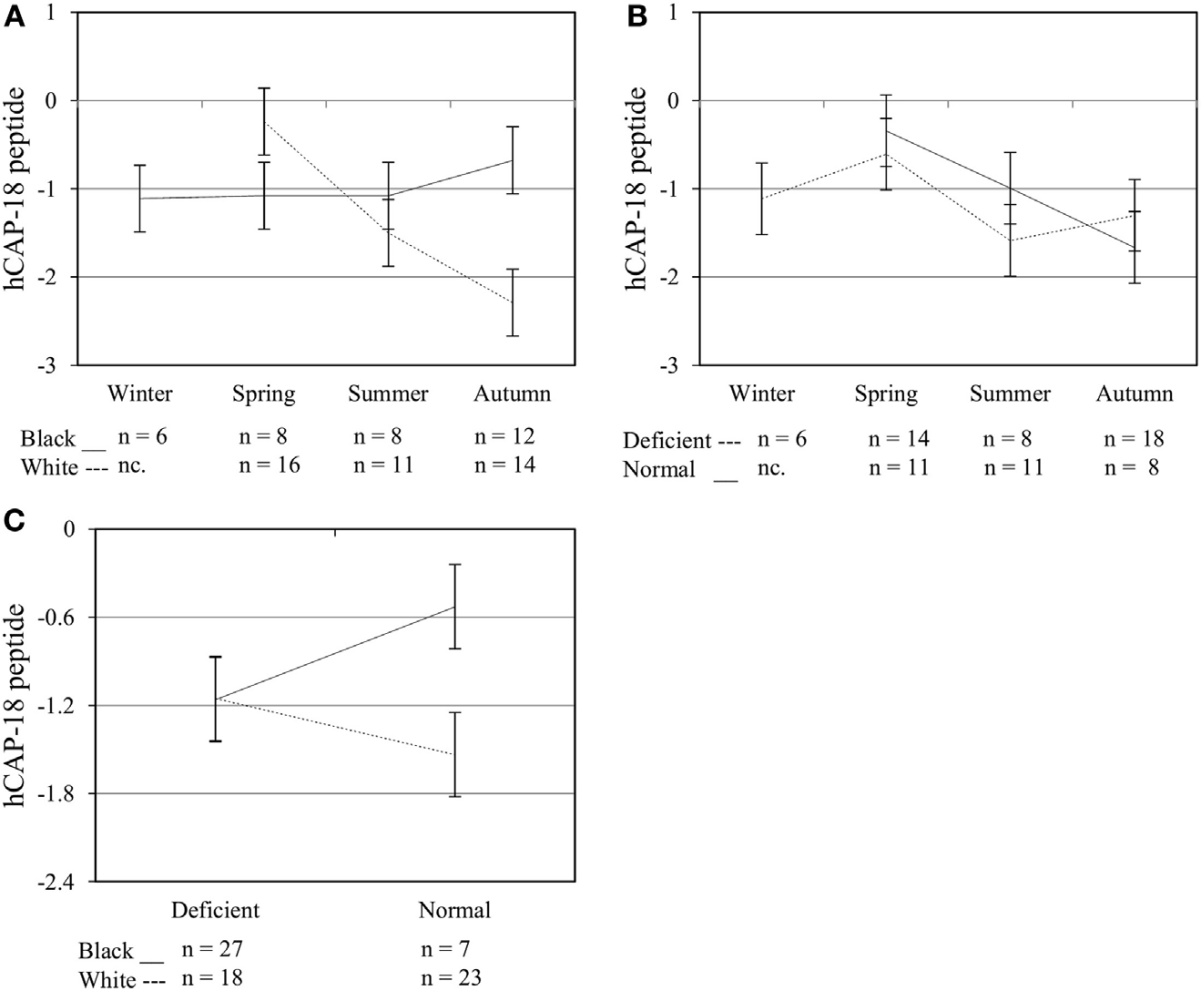
Interaction between season, race, and 25(OH)D_3_ status influence intracellular hCAP-18 peptide level. The line graphs show the interaction effects for hCAP-18 peptide level, quantified by flow cytometry, in monocytes/macrophages from healthy Black (*n* = 34) and White (*n* = 41) South Africans. Season and race [**(A)**, *P* < 0.001], season, and 25(OH)D_3_ status [**(B)**, *P* < 0.010], and race and 25(OH)D_3_ status [**(C)**, *P* < 0.001] interacted significantly to influence hCAP-18 levels. Error bars show the least significant difference at *P* < 0.050. nc. = not collected.

**Figure 7:**
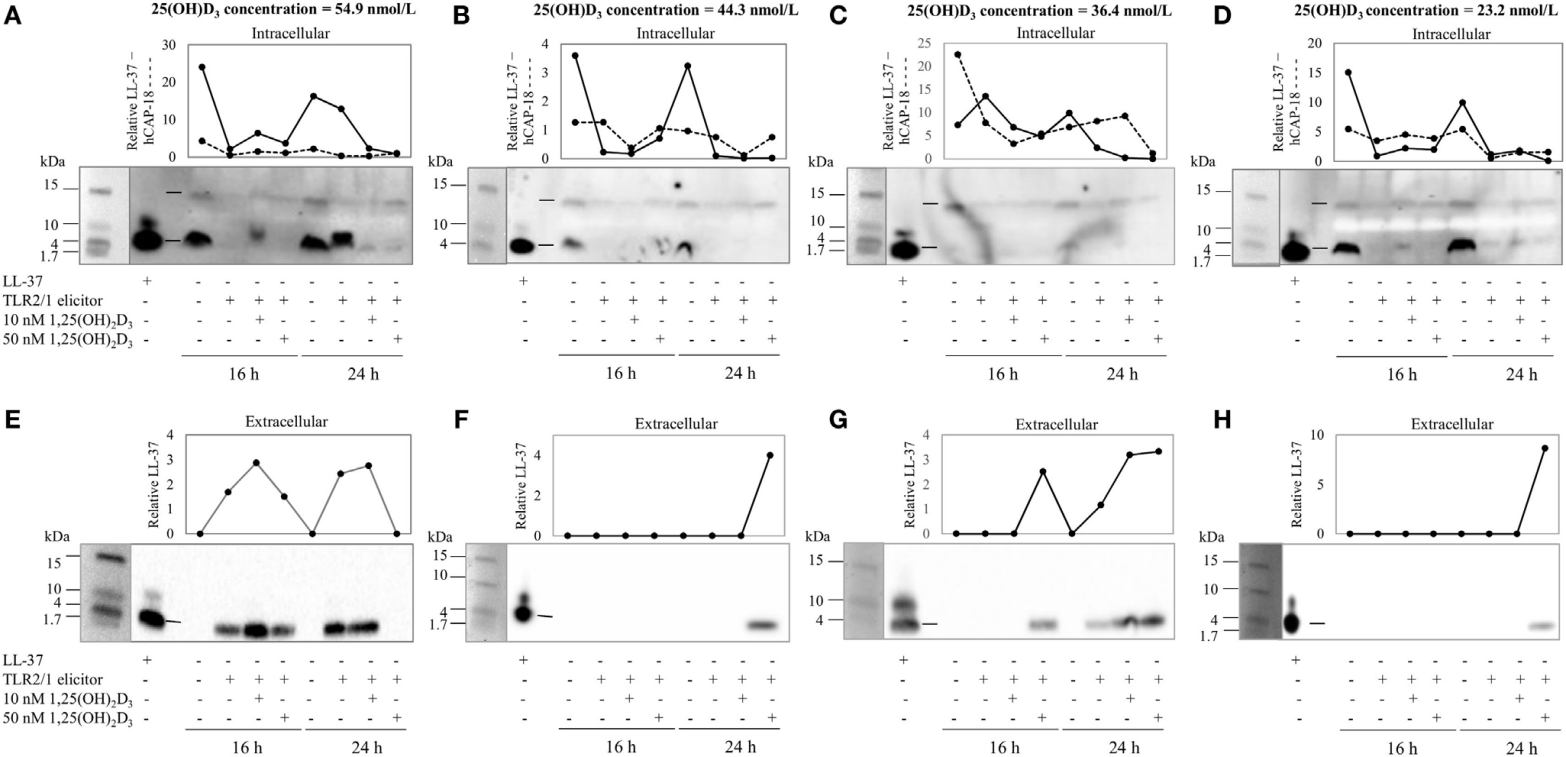
The rate of hCAP-18 processing and secretion is individual-specific and 1,25(OH)_2_D_3_-dependent. The Western blots and accompanying line graphs (densitometry results) show the relative levels of hCAP-18 (15 kDa) and LL-37 (4 kDa) in monocytes/macrophages [intracellular, **(A–D)**] and LL-37 secreted into culture media [extracellular, **(E–H)**] in response to toll-like receptor (TLR) 2/1 elicitation in the absence or presence 10 or 50 nM 1,25(OH)_2_D_3_ at 16 h and 24 h. Four individuals from a study of healthy South Africans (*n* = 20) are shown, representing Blacks and Whites of different 25(OH)D_3_ status [**(A,E)**: 25(OH)D_3_ sufficient White; **(B,F)**: 25(OH)D_3_-deficient White; **(C,G)**: 25(OH)D_3_-deficient Black; and **(D,H)**: 25(OH)D_3_-deficient Black]. Relative protein or peptide level in monocytes/macrophages or culture media (enriched for proteins < 50 kDa through ultrafiltration) was quantified and normalized to total protein loaded (dotted line, cathelin; solid line, LL-37). Single dashes on blots, between lanes for LL-37 standard and control sample, indicate the position of hCAP-18 (15 kDa), without signaling peptide (3 kDa, not detected), and LL-37 (4 kDa) cleaved from cathelin (11 kDa, not detected).

To assess the efficacy of the TLR2/1-VDR signaling regarding functional variables in the pathway (*VDR* mRNA, VDR protein, *CAMP* mRNA, hCAP-18, and *CYP24A1* mRNA), multivariate analysis of the effect of race, 25(OH)D_3_ status, season, and treatment (with/without *in vitro* 1,25(OH)_2_D_3_ supplementation and/or TLR2/1 elicitation) was performed (Table 2). Treatment had a significant main effect on functional variables (Figure 3), while season, race, and 25(OH)D_3_ status showed several complex interactions regarding *VDR* mRNA and VDR protein (Figure 4), *CAMP* mRNA and *CYP24A1* mRNA (Figure 5) and hCAP-18 (Figure 6). TLR2/1 elicitation induced VDR protein (Figure 3B, *P* < 0.001), while 1,25(OH)_2_D_3_ induced *CAMP* mRNA and *CYP24A1* mRNA (Figures 3C,E, *P* < 0.001). Considering interactions, 25(OH)D_3_-deficient Blacks had significantly lower *VDR* mRNA in summer than deficient Whites or Whites and Blacks with a normal 25(OH)D_3_ status (*P* < 0.050, Figure 4A). In contrast, 25(OH)D_3_-deficient Whites had significantly lower *VDR* mRNA in autumn than deficient Blacks or Whites and Blacks with a normal 25(OH)D_3_ status (*P* < 0.050). VDR protein dropped significantly in summer and autumn for Whites and Blacks, respectively, showing a significant race difference in summer (*P* < 0.050, Figure 4B). *CAMP* mRNA increased significantly from summer to autumn in Whites, being significantly higher than Blacks (*P* < 0.050, Figure 5A). Whites with a normal 25(OH)D_3_ status had significantly higher *CYP24A1* mRNA than 25(OH)D_3_-deficient Whites or Blacks and Blacks with a normal 25(OH)D_3_ status (*P* < 0.050, Figure 5B). A notable decrease in hCAP-18 was observed in Whites from spring through summer to autumn being significantly higher in spring and significantly lower in autumn, compared to Blacks (*P* < 0.050, Figure 6A). All individuals with a normal 25(OH)D_3_ status showed a similar, significant decrease in hCAP-18 from spring to autumn (*P* < 0.050, Figure 6B). Blacks with a normal 25(OH)D_3_ status had significantly more hCAP-18, than normal Whites and deficient Blacks or Whites (*P* < 0.050, Figure 6C). To confirm that the intracellular decrease in hCAP-18 in response to seasons with higher 1,25(OH)_2_D_3_ reflects hCAP-18 processing and LL-37 secretion, we performed Western blotting on 20 additional randomly selected healthy Black (*n* = 10) and White (*n* = 10) South Africans. These individuals, collected in winter and for whom no other variables were quantified, were also included in 25(OH)D_3_ quantification (shown in Figure 1). Western blotting showed individual-specific hCAP-18 processing and LL-37 secretion, which depended on 25(OH)D_3_ status, extent of 1,25(OH)_2_D_3_ supplementation, and incubation time (Figure 7). For example, an individual with a sufficient 25(OH)D_3_ status (>50 nmol/L, Figure 7A) had the highest level of intracellular LL-37 under control condition and already secreted LL-37 upon TLR2/1 elicitation at 16 h, and secreted even more with moderate 1,25(OH)_2_D_3_ supplementation (10 nM) or at 24 h. However, with excessive (50 nM) supplementation, the individual secreted less (16 h) or none (24 h). In contrast, 25(OH)D_3_-deficient individuals secreted LL-37 slower and required at least 50 nM of 1,25(OH)_2_D_3_ to secrete at 16 h (Figure 7C). An individual with severe 25(OH)D_3_ deficiency (23.2 nmol/L) secreted LL-37 only after 24 h in the presence of 50 nM 1,25(OH)_2_D_3_ (Figure 7D). It should be noted that secreted LL-37 was undetectably low in 13 of the 20 randomly selected individuals subjected to Western blotting.

**Table 2:** Multivariate main and interaction effects of season, race, 25(OH)D_3_ status, and treatment on the functional variables marking TLR2/1 vitamin D receptor (VDR)-mediated signaling.

	Multivariate *P*-value^a^	Tests of between-subjects				
Independent variable		ln *VDR* mRNA	VDR protein	ln CAMP mRNA	ln hCAP-18 peptide	ln CYP24A1 mRNA
Season	0.000***	0.089	0.003**	0.000***	0.000***	0.000***
Race	0.000***	0.146	0.617	0.000***	0.000***	0.000***
25(OH)D_3_ status	0.135	na.^b^	na.	na.	na.	na.
Treatment	0.000***	0.999	0.000***	0.000***	0.385	0.000***
Season × race	0.000***	0.000***	0.000***	0.010*	0.000***	0.543
Season × 25(OH)D_3_ status	0.000***	0.024*	0.055	0.259	0.001**	0.000***
Season × treatment	1.000	na.	na.	na.	na.	na.
Race × 25(OH)D_3_ status	0.000***	0.866	0.346	0.092	0.000***	0.001**
Race × treatment	0.957	na.	na.	na.	na.	na.
25(OH)D_3_ status × treatment	0.999	na.	na.	na.	na.	na.
Season × race × 25(OH)D_3_ status	0.002**	0.001**	0.848	0.530	0.069	0.012*
Season × race × treatment	1.000	na.	na.	na.	na.	na.
Season × 25(OH)D_3_ status × treatment	1.000	na.	na.	na.	na.	na.
Race × 25(OH)D_3_ status × treatment	0.919	na.	na.	na.	na.	na.
Season × race × 25(OH)D_3_ status × treatment	0.998	na.	na.	na.	na.	na.
Levene’s^c^		0.093	0.121	0.080	0.598	0.004**

^a^Multivariate Pillai’s trace statistic is significant at *P < 0.050, **P < 0.010, and ***P < 0.001.

*^b^na. Not applicable for testing due to multivariate P > 0.050*.

^c^Levene’s Test of Equality of Error Variances is significant at **P < 0.010.

### Correlation Analysis

To further explore the relation between independent variables (UVI, 25(OH)D_3_ concentration, age and regional methylation) and dependent functional variables, following different treatments, correlation analysis was performed (Table 3). UVI correlated moderately with circulating 25(OH)D_3_, which in turn showed a moderate, negative correlation with regional methylation of the *VDR* promoter CGI 1062. Regarding inter-CGI correlations, CGI 1066 and 1060b correlated negatively with CGI 1062, 1061, and 1060a. The significant correlation between 25(OH)D_3_ and methylation was maintained irrespective of season, with partial correlations controlling for seasonal changes in UVI yielding similar correlation coefficients (data not shown). Considering functional variables, regional methylation showed moderate correlation with VDR protein under certain treatments: CGI 1066 and 1060b (positive for all treatments but baseline); 1060a (negative for all treatments but baseline); 1062 and 1061 (negative for 1,25(OH)_2_D_3_ and/or TLR2/1 elicitor). As expected, each functional variable showed correlation between all treatments (not shown). In addition, *VDR* mRNA showed a number of moderate positive correlations with *CAMP* mRNA, across a number of treatments, similarly so, *CAMP* with *CYP24A1* mRNA. Age showed a moderate negative correlation with hCAP-18 peptide with TLR2/1 elicitor + 1,25(OH)_2_D_3_ (*r* = −0.330, *P* < 0.01, *n* = 99).

**Table 3:** Significant correlations between independent environmental variables [season (UVI), plasma 25(OH)D_3_ concentration] and dependent TLR2/1-VDR signaling variables, marking *VDR* expression (*VDR* mRNA, VDR protein) and function (*CAMP* mRNA, *CYP24A1* mRNA, hCAP-18 protein)^a^.

		UVI	Circulating 25(OH)D_3_	All GCIs	CGI 1066	CGI 1062	CGI 1061	CGI 1060	CGI 1060a	CGI 1060b
Circulating 25(OH)D_3_		0.396** (71)								

Regional methylation	All GCIs	ns.	ns.							
	CGI 1066	ns.	ns.	0.478*** (96)						
	CGI 1062	−0.228* (88)	−0.368** (83)	0.552*** (96)	ns.					
	CGI 1061	ns.	−0.287** (80)	0.262** (96)	−0.399*** (96)	0.341** (97)				
	CGI 1060	ns.	ns.	0.657*** (96)	ns.	0.229* (99)	0.327** (97)			
	CGI 1060a	ns.	−0.255* (82)	0.338** (96)	−0.329** (96)	0.497*** (99)	0.648*** (97)	0.435*** (99)		
	CGI 1060b	ns.	0.298** (81)	0.229* (95)	0.388*** (95)	−0.285** (98)	−0.458*** (96)	0.269** (98)	−0.453*** (98)	

ln *VDR* mRNA	Baseline	0.326** (84)	0.223* (78)	ns.	ns.	ns.	ns.	ns.	ns.	ns.
	Control	0.273* (84)	ns.	ns.	ns.	ns.	ns.	ns.	ns.	ns.
	1,25(OH)_2_D_3_	0.307** (83)	0.274* (77)	ns.	ns.	ns.	ns.	ns.	ns.	ns.
	TLR2/1 elicitor	ns.	0.277* (77)	ns.	ns.	ns.	ns.	ns.	ns.	ns.
	TLR2/1 elicitor + 1,25(OH)_2_D_3_	0.246* (84)	0.233* (78)	ns.	ns.	ns.	ns.	ns.	ns.	ns.

VDR protein	Baseline	−0.276** (87)	ns.	ns.	ns.	ns.	ns.	ns.	ns.	ns.
	Control	ns.	ns.	ns.	0.349** (94)	ns.	ns.	ns.	−0.200* (97)	0.206* (96)
	1,25(OH)_2_D_3_	ns.	ns.	ns.	0.296** (95)	−0.204* (99)	ns.	ns.	−0.317** (98)	0.303** (97)
	TLR2/1 elicitor	ns.	ns.	ns.	0.293** (95)	−0.227* (99)	−0.236* (96)	−0.245* (98)	−0.348*** (98)	0.201* (97)
	TLR2/1 elicitor + 1,25(OH)_2_D_3_	ns.	ns.	ns.	0.336** (95)	−0.267** (99)	−0.246* (96)	ns.	−0.362*** (98)	0.364*** (97)

ln *CAMP* mRNA	Baseline	ns.	ns.	ns.	ns.	ns.	ns.	ns.	ns.	ns.
	Control	0.249* (83)	ns.	ns.	ns.	ns.	ns.	ns.	ns.	ns.
	1,25(OH)_2_D_3_	0.308** (84)	ns.	ns.	−0.268* (91)	ns.	ns.	ns.	ns.	ns.
	TLR2/1 elicitor	0.226* (82)	0.246* (77)	ns.	ns.	ns.	ns.	ns.	ns.	ns.
	TLR2/1 elicitor + 1,25(OH)_2_D_3_	0.249* (84)	ns.	ns.	ns.	ns.	ns.	ns.	ns.	ns.

ln hCAP-18 peptide	Baseline	−0.216* (87)	ns.	ns.	ns.	ns.	0.203* (95)	ns.	ns.	−0.202* (96)
	Control	−0.214* (88)	ns.	ns.	ns.	ns.	ns.	ns.	ns.	ns.
	1,25(OH)_2_D_3_	ns.		ns.	ns.	ns.	ns.	ns.	ns.	ns.
	TLR2/1 elicitor	−0.264* (88)	ns.	ns.	ns.	ns.	ns.	ns.	ns.	ns.
	TLR2/1 elicitor + 1,25(OH)_2_D_3_	−0.278** (88)	ns.	ns.	ns.	ns.	ns.	ns.	ns.	ns.

ln *CYP24A1* mRNA	Baseline	ns.	ns.	ns.	ns.	ns.	ns.	ns.	ns.	ns.
	Control	ns.	ns.	ns.	ns.	ns.	ns.	ns.	ns.	ns.
	1,25(OH)_2_D_3_	ns.	ns.	ns.	ns.	ns.	ns.	ns.	ns.	ns.
	TLR2/1 elicitor	ns.	ns.	ns.	−0.251* (91)	ns.	ns.	ns.	ns.	ns.
	TLR2/1 elicitor + 1,25(OH)_2_D_3_	ns.	ns.	ns.	ns.	ns.	ns.	ns.	ns.	ns.

^a^The two-tailed Spearman’s rho correlation coefficients were significant at *P < 0.050, **P < 0.010, and ***P < 0.001. The adjusted significance threshold for multiple comparisons is **P < 0.010. The number of cases included for each test is shown in brackets next to the correlation coefficient for significant correlations.

*CAMP, cathelicidin antimicrobial peptide; CGI, CpG island; CpG, cytosine-phosphate-guanine dinucleotide; CYP24A1, 25-hydroxyvitamin D_3_-24-hydroxylase; ns, not significant; TLR, toll-like receptor; UVI, ultraviolet index; VDR, vitamin D receptor*.

### The Combined Impact of Genetics, Epigenetics, and Environment on TLR2/1-VDR Signaling

To identify the main variables underlying differential levels of *VDR* and downstream targets (*CAMP*, hCAP-18, and *CYP24A1*), the multivariate OPLS-DA statistical method was performed and validated as described (Methods S1.3 in Supplementary Material). Evaluation of the loadings S-plots (Methods S1.3 and Figure S4 in Supplementary Material) and descriptive assessment of the scores space for each model, identified combined effects of genetics, *VDR* methylation, vitamin D status, and UVI on the efficacy of TLR2/1-VDR signaling, as assessed by the level of functional variables produced in response to various treatments. Functional variables were categorized as above/below average and X (independent) variables that significantly (*P* ≤ 0.050) and/or measurably (≥1.5-fold or ≤0.667) discriminate mean values for above/below average response were recorded with their correlation (Table 4). Methylation at CGI 1062, CpG 3 significantly and most notably discriminate mean values for above and below average VDR protein level, particularly with TLR2/1 elicitation. Other sizeable methylation–function interactions observed that were significant and occurred in at least two treatments of a functional variable included 1060 CpG 6 [positive impact on *VDR* mRNA with 1,25(OH)_2_D_3_ supplementation or TLR2/1 elicitation], 1062 CpG 23 (negative impact on hCAP-18 at control and elicitation, with or without supplementation), and CGI 1060a across several neighboring CpGs [1060 CpG 1-5, negative impact for 1,25(OH)_2_D_3_ supplementation, TLR2/1 elicitation or both, clustering by 25(OH)D_3_ status in the absence of supplement and by the *Taq*I *VDR* SNP with/without supplement]. Methylation at CGI 1066, CpG 1-6 also influenced VDR protein level positively following 1,25(OH)_2_D_3_ treatment, with or without TLR2/1 elicitor, showing clustering based on race and SNPs in *VDR* (*Bsm*I, *Apa*I, and *Taq*I), *TLR1* (I602S), and *TIRAP* (S180L), particularly in the presence of supplement. Compared to VDR protein, *CAMP* mRNA level was inversely impacted by CGI 1066, CpG 1-6 methylation, following 1,25(OH)_2_D_3_ treatment, while it clustered only based on 25(OH)D_3_ status and *Taq*I. Methylation of the CpG located at *Taq*I was the only methylation site with significant impact on *VDR* mRNA level following 1,25(OH)_2_D_3_ or TLR2/1 elicitor, but not both. Considering environmental factors, UVI and 25(OH)D_3_ significantly influenced *VDR* mRNA level following treatment with 1,25(OH)_2_D_3_, TLR2/1 elicitor, or both. VDR protein level at baseline and *CAMP* and *CYP24A1* mRNA upon elicitation were significantly influenced by 25(OH)D_3_. 25(OH)D_3_ status and *Taq*I most consistently showed clustering during descriptive assessment of the scores space of computed models. Model construction for hCAP-18 was less favorable and we were unable to identify clustering. Clustering by season, although tested, was not observed.

**Table 4:** Fold change (above/below average means) and correlation for functional variables, significantly and/or prominently, impacted by VDR methylation, 25(OH)D_3_, and UVI, together with population clustering, observed through score space assessment.

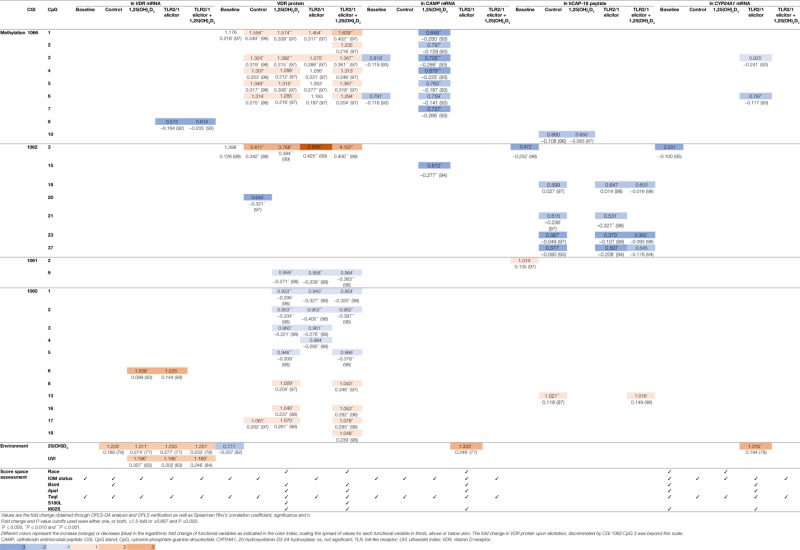

## Discussion

Using a healthy South African cohort, we studied the combined effect of *VDR* methylation, TLR2/1-VDR pathway SNPs, and environment on TLR2/1 signaling and inter-individual variation in the response to vitamin D supplementation.

Results support race-related seasonal variation in 25(OH)D_3_ status (29), though, on average, Blacks were 25(OH)D_3_ deficient irrespective of season (Figure 1). Since TLR2/1-VDR signaling to induce *CAMP* greatly depends on the availability of 1,25(OH)_2_D_3_, Black South Africans may have an overall weaker immune response to bacterial pathogens and may benefit from vitamin D supplementation all year round. However, 25(OH)D_3_, season, and race showed complex interactions that influence TLR2/1-VDR signaling, rendering blanket supplementation presumptuous. For example, not all Blacks in the current study were 25(OH)D_3_ deficient (14% were sufficient). Western blotting of secreted LL-37 showed a decreased LL-37 secretion upon supplementation of 25(OH)D_3_ sufficient individuals (Figure 7), supporting the proposed U-shaped relationship between serum 25(OH)D_3_ and health (30).

As expected for regional methylation of expressed genes (31), *VDR* enhancer (1066) and promoter (1062) CGIs were hypomethylated, while the gene-body CGIs (1061 and 1060) were hypermethylated (Figure 2). Blacks had significantly higher methylation at CGI 1062 and CGI 1060a, but lower methylation at 1060b than Whites. The race-specific variation in methylation observed here agrees with Heyn et al. (32) and Adkins et al. (33) who independently showed genome-wide methylation differences between populations, contributing to natural variation. Similarly, Andraos et al. (34) showed significantly higher methylation levels in the Nigerian Yoruba population compared to European Caucasians at several CpG sites within *VDR* CGI 1060. CGI 1060 spans key features; the 5′ splice site for exon 9 (partly encoding the ligand binding domain for VDR), TaqI/CpG 6 embedded in a putative VDRE (35) and the promoter of an untranslated transcript (AK024830) for which the transcription start site is a few bp downstream of a miRNA-125b target site. Thus, differential methylation within this region of the *VDR* may have profound effects on the expression of *VDR* and subsequent efficacy of the TLR2/1-VDR signaling pathway. Indeed, we have previously shown ethnicity-dependent methylation of *VDR* CGI 1060 to distinguish tuberculosis cases from controls (34).

The significant correlation between *VDR* methylation and plasma 25(OH)D_3_ supports the proposed relationship between vitamin D and the epigenome (36–39). The inverse relationship between vitamin D and *VDR* methylation, especially at the primary promoter-spanning CGI 1062, suggests that in addition to the decrease in ligand, increased promoter methylation may be present in vitamin D-deficient individuals, further dampening the TLR2/1-VDR signal. Thus, vitamin D may interact with the epigenome to influence immune function. Indeed, the higher the methylation at CGI 1062 and 1060a, the less VDR protein is present in response to 1,25(OH)_2_D_3_ supplementation and/or TLR2/1 elicitation (Table 3).

Besides epigenetic differences, SNP frequency distribution for all, except *VDR Bsm*I and *Taq*I, differed significantly between races with large effect sizes observed for *GC* rs7041, *TLR1* A7202G, N248S and I602S, and *VDR* Cdx-2 (Table 1). Similar results were obtained for the 1000 Genomes Project’s YRI and CEU populations, except for *Taq*I and *Bsm*I being significant, but not *Apa*I (Table S2 in Supplementary Material). These striking differences in frequency distribution of disease-associated or functionally relevant SNPs support the likelihood of inter-individual variation in TLR2/1-VDR signaling, response to vitamin D supplementation and immune function. For example, the two *GC* SNPs rs7041 and rs4588 create the three common Gc/DBP isoforms (Gc_1_F, Gc_1_S, and Gc_2_) showing significant geographical- and race-specific distribution patterns. The Gc_1_F alleles (rs7041: T allele, rs4588: C allele) are more common among African-Americans and Africans, while the Gc_1_S alleles (rs7041: G allele, rs4588: C allele) are more common among Europeans (40, 41). Gc_1_F and Gc_1_S have a stronger affinity for 25(OH)D compared to Gc_2_ (42), proposed to deliver 25(OH)D more efficiently to target tissues (43). Gc_1_F/Gc_1_F homozygotes have the lowest DBP level and Gc_1_S/Gc_1_S the highest, yet the bioavailable (unbound or free) 25(OH)D is similar between the isoforms (44). Thus, the efficacy of 25(OH)D_3_ delivery to target cells may be influenced by genetics and may contribute to the differential response to vitamin D supplementation.

The *in vitro* model used confirmed the induction of VDR by TLR2/1 elicitation (5) and ligand dependance of VDR transactivation of *CAMP* and *CYP24A1* (Figure 3). Observed interactions, regarding race, season and 25(OH)D_3_ status (Figures 4–6) supported the observed correlation between UVI and 25(OH)D_3_ status (23), influencing gene expression and hCAP-18 processing; both processes seemingly hampered/delayed in Blacks or 25(OH)D_3_ deficient individuals. This was also observed for LL-37 secretion in Western blot findings (Figure 7). Significant down regulation of VDR protein in Whites in summer likely reflected negative auto-regulation (24) or UVI-mediated miR125b regulation (20–22).

The relation between *VDR* methylation and functional variables (Table 4) was best observed for VDR protein levels, with CpG sites across the enhancer (CGI 1066), promoter (CGI 1062), exon 3 (CGI 1061) and exon 9 (CGI 1060) showing power to discriminate individuals with above average VDR protein levels from those with below average levels. This supports, in part, the univariate correlation of regional methylation observed most commonly with VDR protein (Table 3). The significant, large discriminatory power of CGI 1062 CpG 3 in the primary promoter of *VDR* to distinguish a 5.7-fold above average mean VDR protein level upon elicitation, may relate to the colocation of a binding site for the E2F transcription factor 7, a member of the V$E2FF matrix family (Matrix Library 10.0, Genomatix 2016 (35)), implicated in negative regulation of DNA binding and transcription (45). Comparing the V$E2FF matrix to the sequence around CGI 1062 CpG 3, showed a matrix and core similarity of 1 and 0.875, respectively, with high conservation across the CpG 3 cytosine-guanine dinucleotide that forms part of the matrix core. Notably, a CpG-ruinous SNPs (C/G) in the second position of the dinucleotide, unique to Africans (5% “C,” 1000 Genomes Browser), have been reported. The positive correlation between VDR protein level and methylation at CGI 1062 CpG 3 may support methylation-sensitive suppressor activity, alleviated by DNA methylation. The positive impact of CGI 1060 CpG 6 methylation, possible only when *Taq*I is “C,” seen for VDR mRNA with supplementation or elicitation, support a TB case control finding from our laboratory showing concomitant decreased methylation of CGI 1060a associating with protection from TB (34) and correlating with increased VDR levels in the current study (Table 3). The prominence of *Taq*I-based clustering across all variables and treatments, except for hCAP-18, further confirms the importance of this SNP, commonly found associated with diverse diseases and first reported to be associated with infectious disease by Bellamy et al. (46). Pam_3_CSK_4_ is a strong activator of NF-κB1 (47) and the negative impact of methylation at 1062 CpG 23 on hCAP-18 may relate to its location adjacent to an NF-κB1 and SP-1 binding site in the primary promoter of *VDR*.

**Figure 8:**
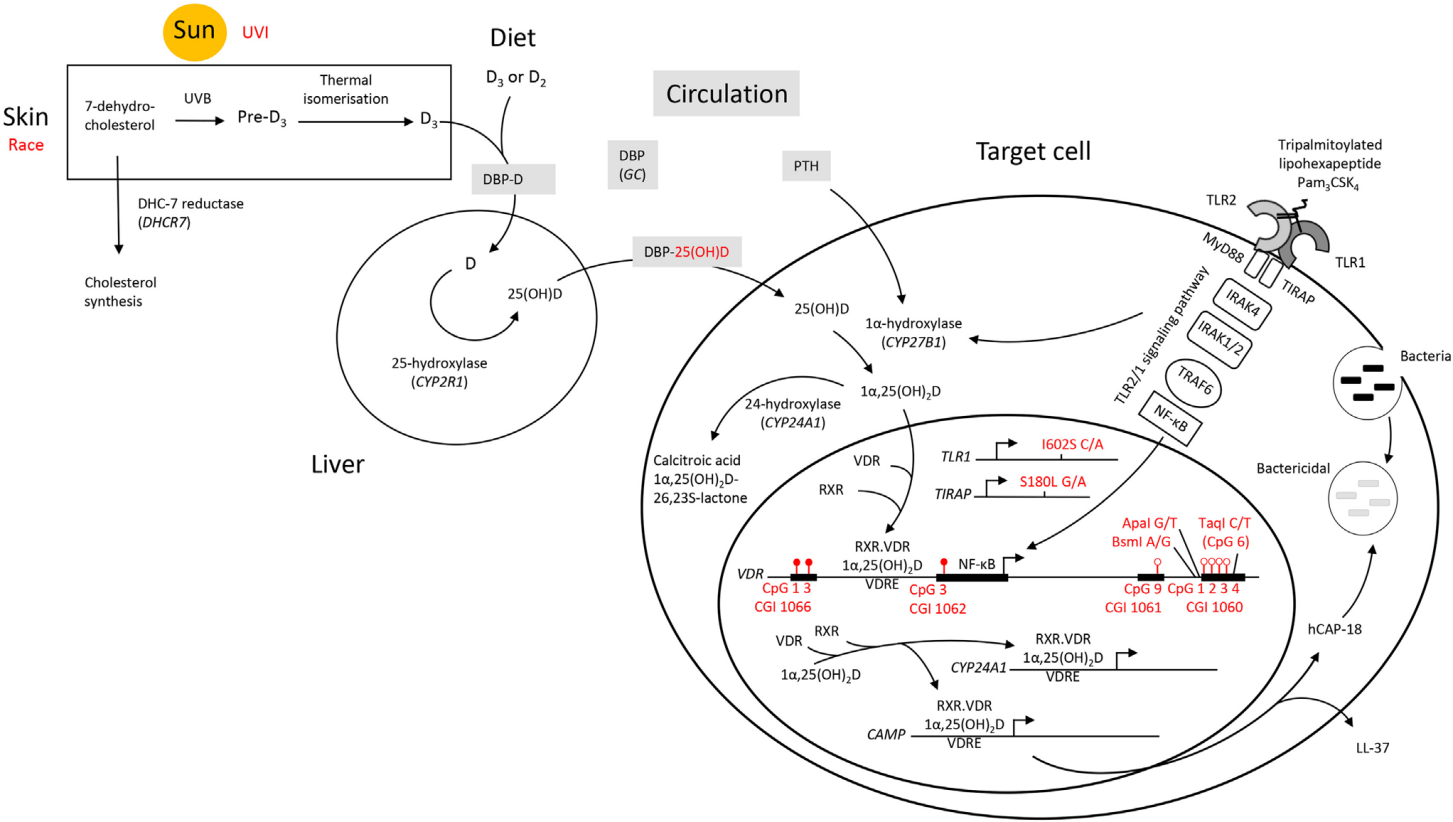
A schematic diagram summarizing factors influencing the signaling efficacy of the TLR2/1-VDR pathway in response to TLR2/1 elicitation. Pam_3_CSK_4_ (Pam_3_CysSerLys_4_), a synthetic tripalmitoylated lipopeptide, mimics the acylated amino terminus of bacterial lipoproteins and is recognized by TLR2, cooperating with TLR1 to induce signaling that activates the pro-inflammatory transcription factor NF-κB, inducing *CYP27B1* and *VDR*. Factors found to influence VDR expression are indicated in red. UVI correlated with 25(OH)D_3_, known to autoregulate *VDR*. Individuals who responded with an above average level of VDR protein upon TLR2/1 elicitation were hypermethylated at CGI 1066 (CpG 1 and 3) and CGI 1062 (CpG 3), while hypomethylated at CGI 1061 (CpG 9) and CGI 1060 (CpG 1–4). With *in vitro* 1,25(OH)_2_D_3_ supplementation CGI 1062 CpG 3 was slightly less discriminatory upon TLR2/1 elicitation, but remained a significant variable to discriminate above/below average VDR protein level. However, with supplementation the population clustered by race and all genetic variants indicated (*TLR1* SNP I602S, *TIRAP* SNP S180L, and *VDR* SNPs *Bsm*I, *Apa*I, and *Taq*I, see Table 4), compared to clustering by vitamin D status and *Taq*I alone, without supplementation. Methylation at CpG 6 of CGI 1060 (dictated by *Taq*I, with which it overlaps) together with UVI and circulating 25(OH)D_3_, significantly discriminated a 1.2-1.5-fold change in VDR mRNA with supplement or elicitation. Abbreviations: *CAMP*, cathelicidin antimicrobial peptide; CGI, CpG island; CpG, cytosine-phosphate-guanine dinucleotide; CYP, cytochrome P-450 enzyme; *CYP24A1*, 25-hydroxyvitamin D_3_-24-hydroxylase; D_2_, ergocalciferol; D_3_, cholecalciferol; DBP, vitamin D binding protein; *GC*, group component or gene encoding DBP; hCAP-18, human cathelicidin antimicrobial peptide 18; LL-37, cathelicidin antimicrobial peptide fragment; 1,25(OH)_2_D_3_ 1,25-dihydroxycholecalciferol; 25(OH)D_3_, 25-hydroxycholecalciferol; NF-κB, nuclear factor kappa B; Pam_3_CSK_4_, synthetic tripalmitoylated lipohexapeptide, PTH parathyroid hormone; RXR retinoid X receptor; *TIRAP*, TIR, toll-interleukin 1 receptor domain-containing adaptor protein; TLR, toll-like receptor; UVB, ultraviolet B rays; UVI, ultraviolet index; *VDR*, vitamin D receptor gene; VDR, vitamin D receptor protein; VDRE, vitamin D receptor element.

Overall, it appears that individuals who respond with an above average level of VDR protein upon TLR2/1 elicitation display hypermethylation at CGI 1066 CpG 1 and 3, as well as at CGI 1062 CpG 3, while displaying hypomethylation at CGI 1061 CpG 9 and CGI 1060a CpG 1-4 (Figure 8). While the *VDR* SNP *Taq*I and 25(OH)D_3_ influenced the variation within the study population with elicitation, without 1,25(OH)_2_D_3_ supplementation, 25(OH)D_3_ status was no longer identified as a contributing factor upon 1,25(OH)_2_D_3_ supplementation. Moreover, the effect of several SNPs became apparent only in the presence 1,25(OH)_2_D_3_ supplementation. This suggests that 25(OH)D_3_ status may have a larger effect on TLR2/1, VDR-mediated signaling than these genetic variables.

Taken together, results presented here provide support for multifactorial regulation of VDR-mediated TLR2/1 signaling, involving interaction between environment, epigenetics, and genetics. UVI influences 25(OH)D_3_ status, which regulates VDR expression through *VDR* methylation, while enhancing the extent and rate of VDR transactivation of *CAMP* encoding the antimicrobial peptide hCAP-18. The complex interaction between these factors may shed further light on the disparity in infectious diseases across the globe.

## Ethics Statement

In accordance with the Declaration of Helsinki, ethical clearance was obtained from the South African National Blood Service (SANBS) and the Faculty of Science, University of Johannesburg. After informed consent, the SANBS collected blood from randomly selected healthy Black (*n* = 50; age 17–62 years; 25 males and 25 females) and White (*n* = 50; age 17–69; 25 males and 25 females) South Africans living in Gauteng, SA.

## Author Contributions

LB designed the study; VM, DS, FA, and TJ acquired the data; VM, FT, and LB analyzed the data; VM and LB wrote the manuscript; DS, FA, TJ, and FT revised the manuscript; and all authors approved the final version to be published and agree to be accountable for all aspects of the work.

## Conflict of Interest Statement

The authors declare that the research was conducted in the absence of any commercial or financial relationships that could be construed as a potential conflict of interest.

## Abbreviations

*CAMP*, cathelicidin antimicrobial peptide; CGI, CpG island; CpG, Cytosine-phosphate-guanine dinucleotide; CYP cytochrome P-450 enzyme; *CYP24A1*, 25-hydroxyvitamin D_3_-24-hydroxylase; DBP, vitamin D-binding protein; *GC*, group-specific component; hCAP-18, human cathelicidin antimicrobial peptide 18 kDa; IOM, Institute of Medicine; LL-37, cathelicidin antimicrobial peptide fragment; 1,25(OH)_2_D_3_, 1,25-dihydroxycholecalciferol; 25(OH)D_3_, 25-hydroxycholecalciferol; NF-κB, nuclear factor kappa B; OPLS-DA, Orthogonal projections to latent structures discriminant analysis; Pam_3_CSK_4_, synthetic triacylated lipopeptide; N-palmitoyl-S-[2,3-bis(palmitoyloxy)-(*2RS*)-propyl]-[*R*]-cysteinyl-[*S*]-seryl-[*S*]-lysyl-[*S*]-lysyl-[*S*]-lysyl-[S]-lysyl × 3HCL; SANBS, South African National Blood Service; SNP, single-nucleotide polymorphism; *TiRAP*, toll-interleukin 1 receptor (TIR) domain-containing adaptor protein; TLR, toll-like receptor; UVB, ultraviolet B rays; UVI, ultraviolet index; *VDR*, vitamin D receptor.
