# Supplementary material for: Methylation of the Vitamin D Receptor (VDR) Gene, Together with Genetic Variation, Race, and Environment Influence the Signaling Efficacy of the Toll-Like Receptor 2/1-VDR Pathway
Source: Front Immunol. 2017 Sep 11;8:1048. doi: 10.3389/fimmu.2017.01048 (PMC5603903; doi:10.3389/fimmu.2017.01048)
Supplement: Supplementary file 1 [file data_sheet_1.docx]

***Supplementary Material***

**Methylation of the vitamin D receptor (VDR) gene, together with genetic variation, race and environment influence the signaling efficacy of the TLR2/1-VDR pathway.**

Vanessa Meyer^1^, Donovan Sean Saccone^1^, Fidele Tugizimana^1^, Furaha Florence Asani^1^, Tamsyn Jacki Jeffery^1^, Liza Bornman^1^*

^1^Department of Biochemistry, University of Johannesburg, Auckland Park, Johannesburg, South Africa.

*Correspondence to Prof Liza Bornman: lizab@uj.ac.za.

1. **Supplementary methods**
   1. **Bioinformatics workflow**

To identify loci of putative function in the VDR pathway, with the potential to influence gene regulation through genetic and/or epigenetic mechanisms, a comprehensive bioinformatic workflow was developed (Figure S1).

*1.1.1. Defining VDR regulatory regions*

Regulatory regions were defined to include promoters, enhancers and CGIs and were identified using a combination of bioinformatic tools and literature searches. Promoter coordinates were confirmed using Genomatix Software Suite (v2.4) Gene2Promoter tool, including gold standard or experimentally verified promoters. Enhancers were identified by literature search, with “*VDR* enhancers” as search term in NCBI PubMed (<http://www.ncbi.nlm.nih.gov/pubmed/>), including publications between 2006 and 2012. Enhancer coordinates (hg19) were ultimately derived from Zella et al. (2010) [1]. However, the coordinates of the S1 enhancer were expanded downstream to include an additional region found to bind VDR [1], and renamed S1+. Bona fide CGIs were identified on the University of California Santa Cruz (UCSC) Human (*Homo sapiens*) Genome Browser Gateway (<http://genome.ucsc.edu/cgi-bin/hgGateway>) using the track-mapping algorithm. All other CGIs were identified using the CpG Island Searcher tool (<http://cpgislands.usc.edu/>). Parameters for % GC (55%), observed CpG/expected CpG (0.65) and minimum gap between adjacent islands (100 bp) were set as prescribed [2]. The minimum length of an island parameter was set at 200 bp. The genomic coordinates resulting from the analyses of the three regulatory element types were combined and will be referred to as ‘regulatory regions’.

*1.1.2. Identification of genetic and epigenetic features in regulatory regions*

*1.1.2.1. Genetics*

To identify SNPs that may influence gene regulation by destroying or creating transcription factor binding sites (TFBSs), rs (RefSNP) numbers of SNPs in regulatory regions were retrieved from dbSNP (<http://www.ncbi.nlm.nih.gov/projects/SNP/>, build 135) in FASTA format. These files were uploaded to Genomatix (v2.4) and submitted to the SNPInspector tool to identify putative TFBSs affected by SNPs. The ‘general core promoter elements’ and ‘vertebrate transcription factor binding site matrices’ were selected as filters. SNPs in intron-exon borders were identified by querying Entrez (<http://www.ncbi.nlm.nih.gov/snp?db=snp>, build 135) with the hg19 genomic coordinates of the regulatory regions as limiting factors for the results. Disease-associated SNPs in the *VDR* and other pathway genes were identified by literature search on PubMed using the gene name (e.g. ‘TLR1’), ‘polymorphism’, ‘SNP’, ‘disease’ and ‘association’ as search terms. Furthermore, the Online Mendelian Inheritance in Man (OMIM) database (<http://www.ncbi.nlm.nih.gov/omim>) was queried and results scanned for relevant disease-associated SNPs. To identify the most conserved regions of the gene, a publicly available track; 28-way most conserved elements, was uploaded onto the hg18 build version of the UCSC genome browser. Genomic coordinates of the most conserved regions occurring in the fourteen placental mammal species and the ten vertebrate species sub-tracks combined, were recorded and used in downstream analyses.

*1.1.2.2. Epigenetics*

DNA methylation, histone modifications and ncRNA were analyzed as possible epigenetic modulators of gene regulation. Cell-specific DNA methylation patterns were analyzed using available relevant cell lines data (GM12878 and K562) from the reduced representation bisulfite sequencing method [3] and the Methyl 450K bead array method. All CpG sites in intron-exon borders were determined by scrutiny of the sequences. Histone modification marks for H_3_K_4_me_1_, H_3_K_4_me_3_, H_3_K_27_ac and H_3_K_27_me_3_ as determined by ChIP-seq were retrieved from ENCODE. The coordinates of strong signal peaks were recorded for use. Where possible, data for these marks were collected from both the Broad Institute and the SYDH (Stanford/Yale/University of Southern California/Harvard) group. DNaseI hypersensitivity clusters were retrieved from ENCODE. The coordinates of miRNA target sites were determined using the TargetScan miRNA Regulatory Sites track. The data were uploaded onto the UCSC genome browser as publicly available tracks.

*1.1.2.3. Genetic-epigenetic interaction*

All regulatory region SNPs were scanned for those creating or destroying CpG sites. SNPs in miRNA target sites were identified by submitting their coordinates as limitations on Entrez (build 135). Evolutionarily conserved CGIs were uploaded onto the UCSC genome browser as the Weizmann Evolutionary CpG Islands track, lifted over from the hg18 build of the human genome.

*1.1.3. Development and implementation of a custom weighted scoring system*

All data collected via *in silico* analysis required representation in a way that allowed efficient cross-referencing of features with the potential to alter expression. This was accomplished by creating separate browser extensible data (BED) format files containing coordinates for each type of regulatory region or feature. These files were uploaded onto the UCSC genome browser as custom tracks and displayed simultaneously with all selected publicly available tracks (Figure S2) at the coordinates chr12:48,225,312-48,347,370 (GRCh37/hg19). A custom weighted scoring system was devised by assigning specific values or points to each genetic and epigenetic feature (Table S1). Where many features clustered and overlapped, particularly those important to gene regulation, small regions (± 500 bp) were selected by scrutinizing the resulting UCSC track images. For all features except CTCF binding, histone modification marks and ENCODE DNA methylation data, points were awarded per instance of a specific feature within the region being scored. For the CTCF binding and histone modification marks, the presence of the feature in the region would earn only one instance of points, regardless of occurring multiple times in the same region, in multiple cell lines, or from different research groups. Regions showing a high number of multiple features were examined more closely. Regions of around 150 bp (no more than 200 bp) *within* each of these ± 500 bp regions were selected to include as many features as possible, with preference given to higher scoring features. 19 key regions were identified which were deemed potentially valuable to genotype and epigenotype, and were subjected to the custom scoring system. The four highest scoring regions, as well as the highest scoring region in an enhancer (the 7^th^ highest), were selected for downstream epigenotyping. A summary of the all the *VDR* CGIs, in context of other regulatory elements, is given in Saccone et al. (2015) [4].


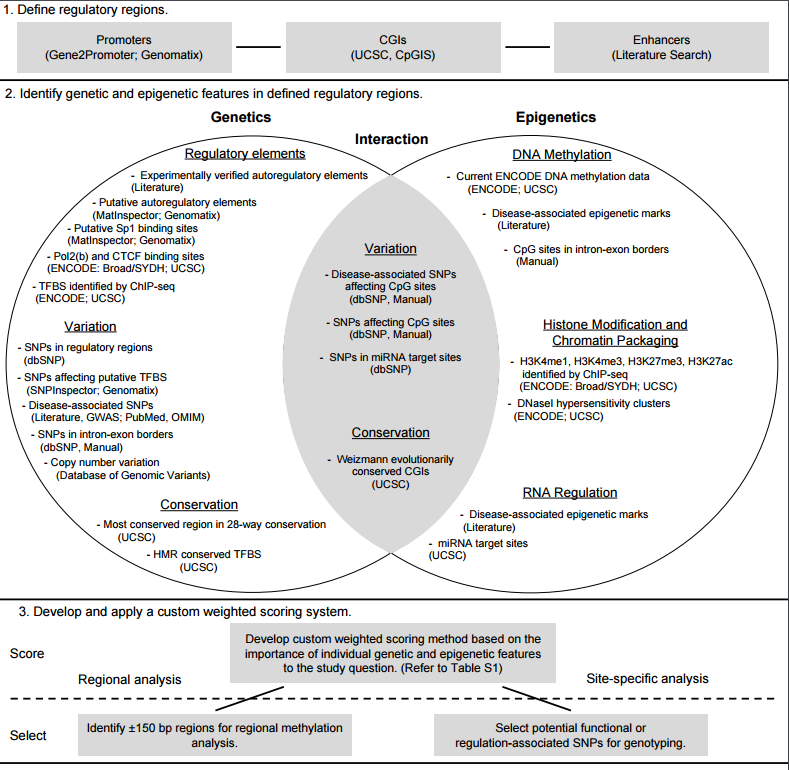


**Figure S1. A** **bioinformatic workflow to identify loci of putative function that potentially influence gene regulation through genetic and/or epigenetic mechanisms.** The diagram shows a workflow for selection of regions with highest potential to underlie differential gene expression. Regulatory regions were defined by identifying promoters, CGIs and enhancers. Within regulatory regions, genetic and epigenetic features were identified. A custom weighted scoring system was designed and regions were scored accordingly, with the highest scoring regions selected for geno- and epigenotyping. *Abbreviations*. CGI: CpG island, HMR: human mouse rat, TFBS: transcription factor binding site(s), ChIP: chromatin immunoprecipitation, GWAS: genome-wide association study.

**(A)**

**
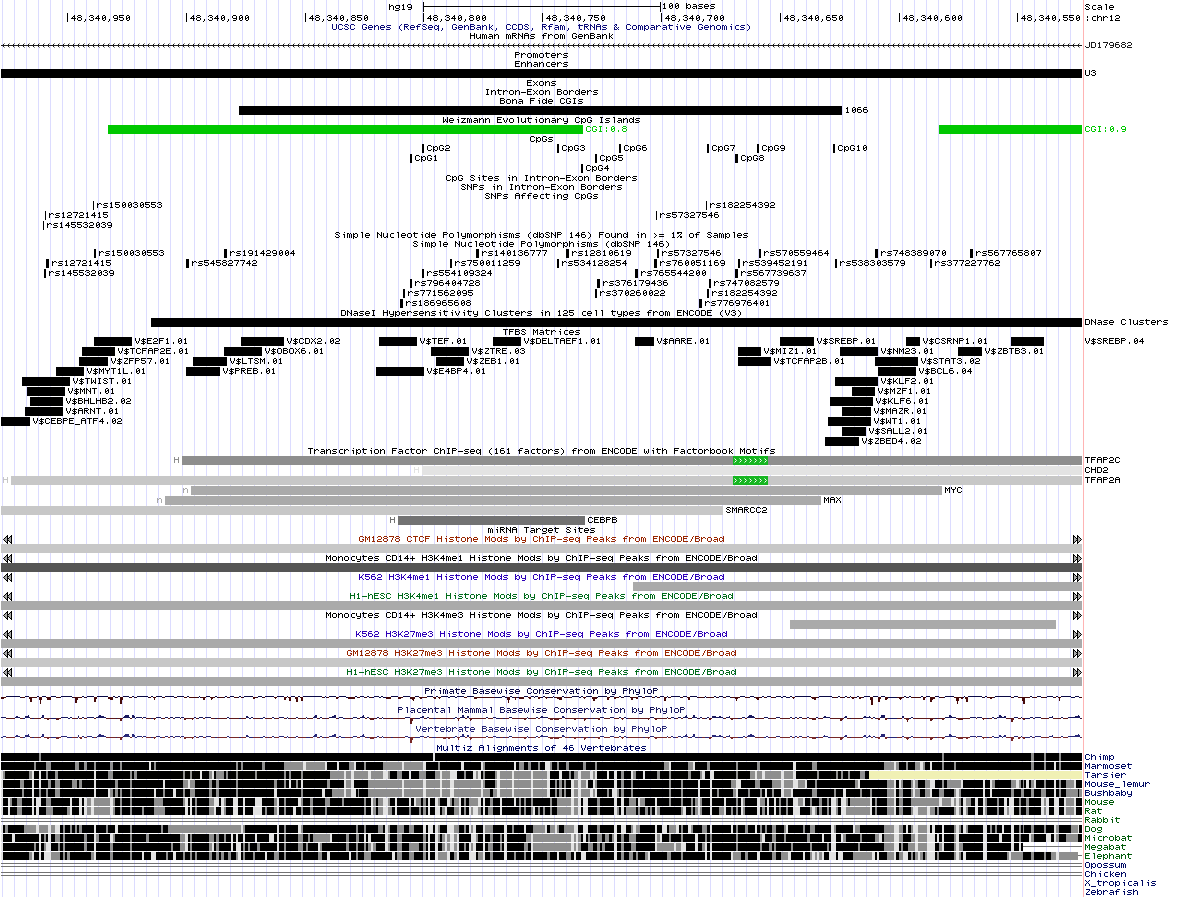
**

**(B)**


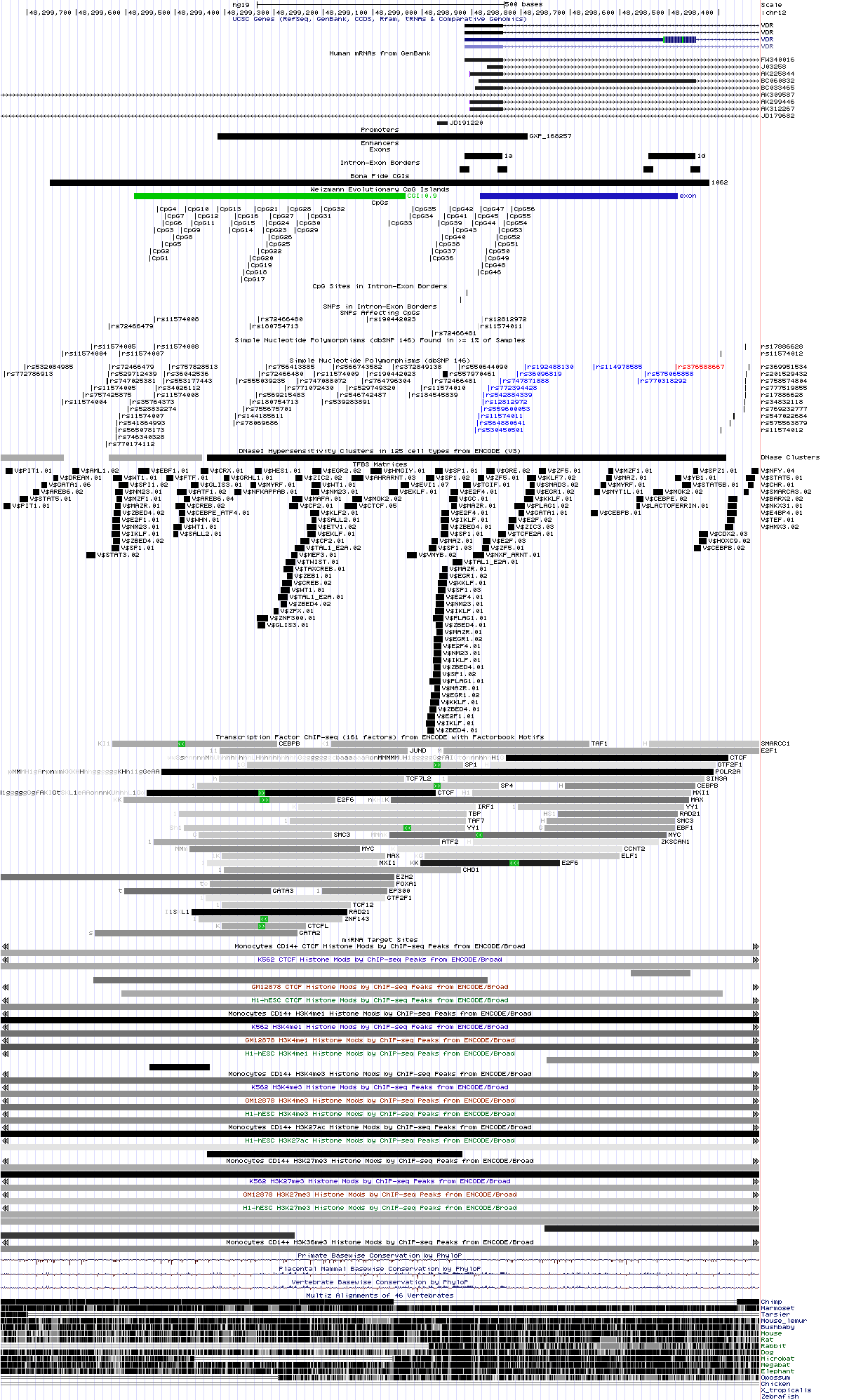


**(C)**


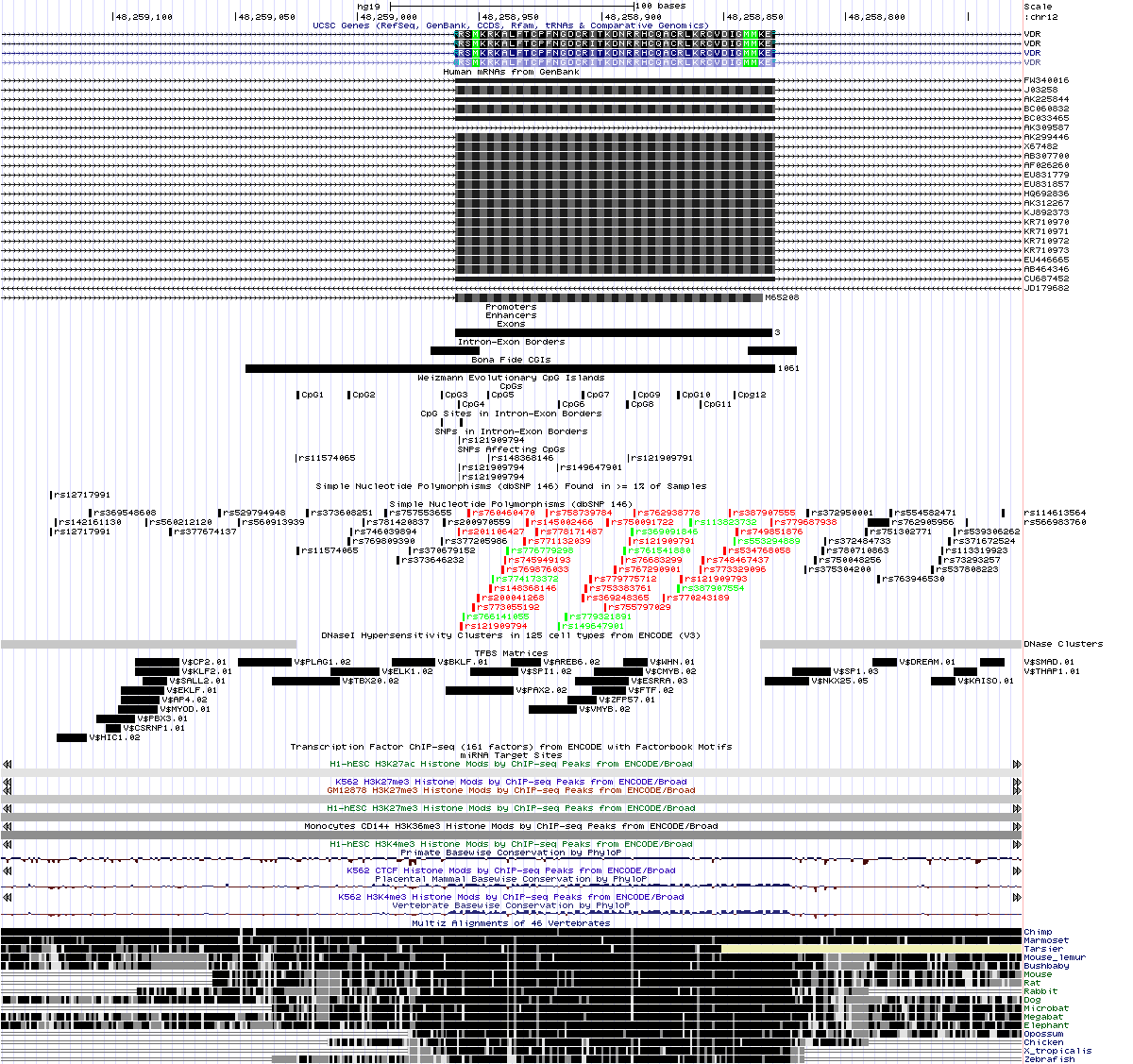


**(D)**

**
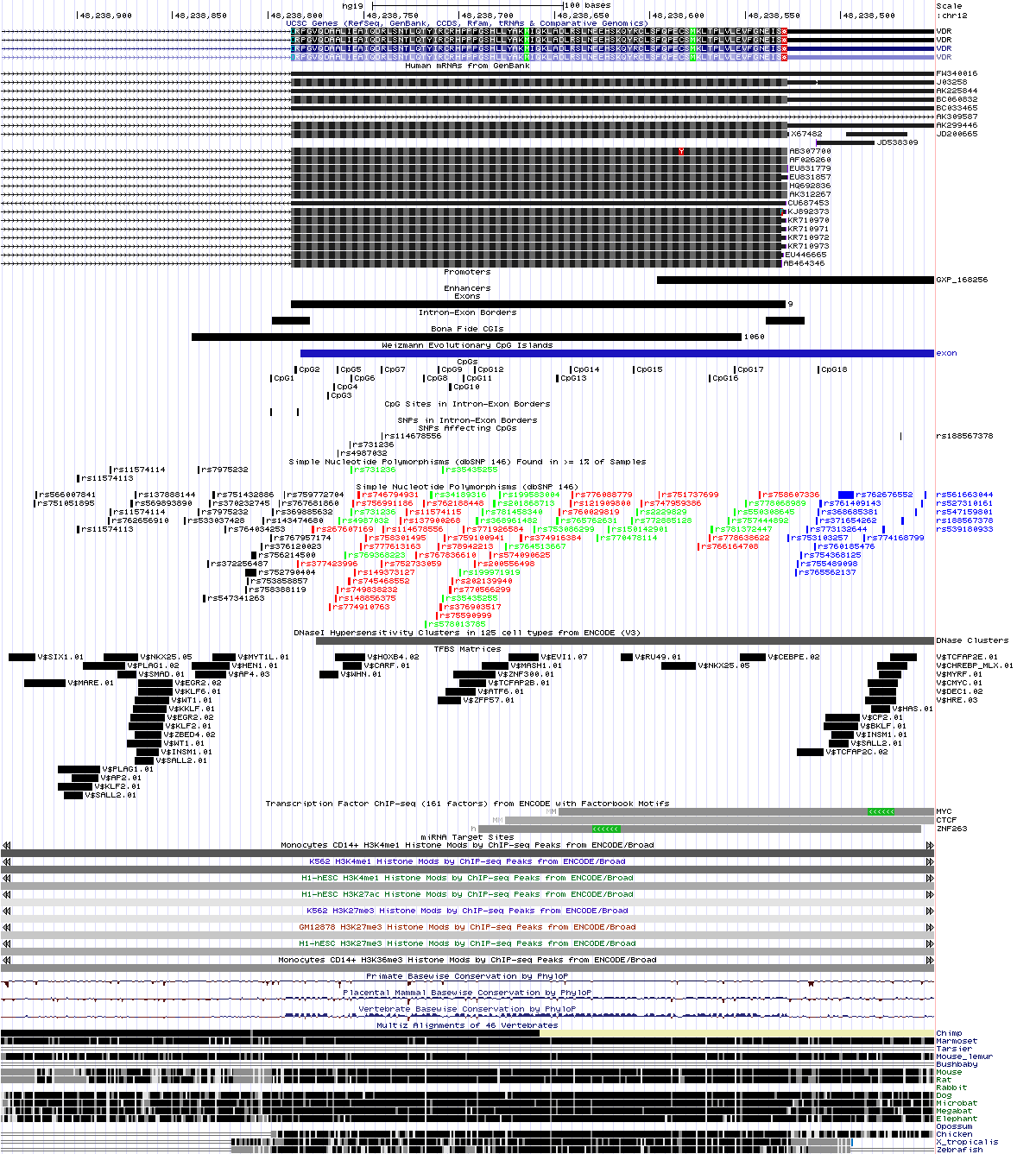
**

**Figure S2**. **Genetic and regulatory annotation of key *VDR* CpG islands (CGIs).** The UCSC Genome Browser images show the CpG sites epigenotyped in CGI 1066 (A, chr12:48340524-48340978; hg19), 1062 (B, chr12:48298219-48299753; hg19), 1061 (C, chr12:48258729-48259146; hg19) and 1060 (D, chr12:48238452-48238940; hg19) in relation to noteworthy genetic, epigenetic and putative regulatory features within the region of the CGI (+100 bp up- and downstream). Tracks for key regulatory features such as promoters and enhancers are included. Human mRNA transcripts (Genbank) are displayed along with exons and their intron-exon borders. Genetic variation is presented in tracks including all SNPs (bdSNP build 146), SNPs with MAF > 1%, SNPs affecting CpG sites, and SNPs in intron-exon borders. SNPs (dbSNP 146) in black are intronic, red are non-synonymous coding SNPs, green are synonymous coding SNPs, and blue are untranslated SNPs. Regulatory control is addressed by tracks for DNaseI hypersensitivity clusters (ENCODE V3), putative TFBS (Genomatix MatInspector Professional 8.21) and transcription factor ChIP-seq (161 factors; ENCODE with Factorbook motifs). Epigenetic factors are represented by tracks for Weizmann evolutionary CGIs, miRNA target sites and histone modifications from ENCODE/Broad Institute (CTCF, H3K4me1, H3K4me3, H3K27ac, H3K27me3, H3K36me3 in the following cells: GM12878, H1-hESC, K562 and primary monocytes). PhyloP assessed conservation using tracks for base wise conservation in primates, placental mammals and vertebrates, as well as tracks for Multiz alignments of common model vertebrates, specifically including nocturnal mammals. For Weizmann evolutionary CGIs, green bars represent CGIs with low rates of C→T deamination, and are typically unmethylated, whereas blue bars represent CGIs overlapping exons.

**Table S1. Point scores assigned to individual genetic and epigenetic features used as region selection criteria tracks on the UCSC Genome Browser.**

| **Feature used as region selection criterion** | **Points assigned to each feature** |
| --- | --- |
| Disease-associated SNPs Affecting CpG Sites | 7 |
| Disease-associated SNPs | 6 |
| Disease-associated Epigenetic Marks |  |
| Weizmann Evolutionary CpG Islands |  |
| ENCODE DNA Methylation |  |
| SNPs Affecting CpG Sites |  |
| Bona Fide CGIs | 5 |
| CpG Sites in Intron-Exon Borders |  |
| SNPs in miRNA Target Sites |  |
| General CGIs | 4 |
| Promoters |  |
| Enhancers |  |
| 28-way Conservation |  |
| SNPs in Intron-Exon Borders |  |
| miRNA Target Sites |  |
| H3K4me1 in PBMCs | 3 |
| H3K4me3 in PBMCs |  |
| H3K27me3 in PBMCs |  |
| Transcription Factor ChIP-seq (methylation sensitive TFs) |  |
| Pol2(b) in K562 Cell Line |  |
| DNaseI Hypersensitivity Clusters |  |
| Exons |  |
| Putative VDREs |  |
| Putative SP1 Binding Sites |  |
| SNPs Affecting Putative TFBS |  |
| H3K27me3 in K562 & GM12878 Cell Lines | 2 |
| CTCF in K562 & GM12878 Cell Lines |  |
| HMR Conserved Transcription Factor Binding Sites |  |
| H3K4me1 in K562 GM12878 Cell Lines | 1 |
| H3K4me3 in K562 & GM12878 Cell Lines |  |
| H3K27ac in K562 & GM12878 Cell Lines |  |
| Transcription Factor ChIP-seq (non-methylation sensitive TFs) |  |

- 1. **Western blotting**

hCAP-18 preproprotein, with a monoisotopic theoretical Mw of 19.3 kDa (ExPASy, Compute pI/Mw tool), comprises a signal peptide (3.3 kDa), conserved cathelin domain (11.5 kDa) and the enzymatically released LL-37 antimicrobial peptide domain (4.5 kDa). To assess hCAP-18 proprotein processing into cathelin and LL-37, and secretion of the latter, cell lysates and cell culture supernatants (media) were analyzed by Western blotting according to the Bio-Rad V3 workflow. Monocyte/macrophages (4 x 10^6^) suspended in 30 µl Laemmli sample buffer (Bio-Rad, Johannesburg, South Africa) were sonicated (10 s) and boiled at 100°C (10 min). Potentially secreted protein and/or peptide were enriched from cell culture supernatant by centrifuging (40 min at 3 000 x *g*) samples through a Vivaspin 20 ™ ultrafiltration column with a molecular weight cut-off of 50 kDa (Sartorius Stedim Biotech, Goettingen, Germany). Proteins and peptides in the filtrate were precipitated with trichloroacetic acid (Merck, Modderfontein, South Africa), dissolved in 20 µl Laemmli sample buffer and boiled at 100°C (10 min). Proteins or peptides in cell lysate (60 µg total protein) or enriched cell culture supernatant (8 µg total protein) were separated through electrophoresis (TGX-Precast stain-free 8-16% gradient gels), alongside 5 µl Spectra Multicolor Low Range Protein Ladder (Thermo Fisher Scientific Inc., Waltham, MA) and purified LL-37 standard (25 ng, Hycult Biotech, Uden, The Netherlands). The primary antibody (mouse IgG_1_ anti-human hCAP-18 antibody; Abcam, Cambridge, United Kingdom, Cat. No. OSX-12) was used at a 1:500 dilution in 2.5% non-fat milk and allowed to bind overnight, shaking at 4°C. The secondary antibody (goat anti-mouse IgG antibody conjugated to horseradish peroxidase; Abcam, Cambridge, United Kingdom, Cat. No. ab20043) was used at a 1:5 000 dilution in 2.5% non-fat milk and allowed to bind for 1 h. Band intensities were quantified by densitometry and normalized against the total protein loaded to facilitate comparison between lanes representing different treatments.

- 1. **Orthogonal projections to latent structures discriminant analysis (OPLS-DA)**

To understand and explain the multivariate relationships in the centered X-data (genetic, epigenetic and environmental factors), with regards to the measured levels of functional variables (*VDR* mRNA, VDR protein, *CAMP* mRNA, hCAP-18 and *CYP24A1* mRNA) in the TLR2/1-VDR signaling pathway, under the five treatments (Baseline, Control, 1,25(OH)_2_D_3_ in vitro supplement, TLR2/1 elicitor and TLR2/1 elicitor + 1,25(OH)_2_D_3_), OPLS-DA modelling was applied. OPLS-DA is an extension of the supervised partial least square-discriminant analysis (PLS-DA) regression method, featuring an integrated orthogonal signal correction (OSC)-filtering method; and as such OPLS-DA modelling has added interpretational and discriminatory benefits compared to PLS-DA. In simple mathematical terms, OPLS-DA uses information in the Y matrix to decompose the X matrix into blocks of structured variation correlated to and orthogonal to Y, respectively explaining predictive and non-predictive (orthogonal) variation [5, 6]. For the OPLS-DA modelling, data input included race, sex, blood group (Rhesus D and ABO), age group (twenties, thirties, etc.), 25(OH)D_3_ status, TLR2/1-VDR pathway SNP genotypes, UVI and site-specific methylation at all CpGs.

Thus, the computed models comprised 1 predictive and 1 orthogonal component, and were validated to assess the robustness, predictive ability, reliability and significance. The model fit (R^2^X) and predictive power (Q^2^) metrics, estimated using 7-fold cross-validation, provided a quantitative measure of the goodness of fit and the predicted variation by the models, respectively. Analysis of variance of cross-validated predictive residuals (CV-ANOVA) assessed the reliability of generated models. The response permutation test was applied and none of the permutated models (n = 100) performed better than the original models in separating classes. Furthermore, the evaluation of receiver operator characteristic (ROC) curves showed that the computed models were good binary classifiers (e.g. area under the curve = 0.728, Figure S3). For discriminant variable selection from continuous data in the model, the OPLS-DA loading S-plots were evaluated. The S-plot is used to visualize both the covariance and the correlation structure between the X-variables and the predictive score t[1]; and it is a scatter plot of the p[1] vs. p(corr)[1] vectors of the predictive component [5, 6]. Thus, to avoid over interpretation of the model and variable selection bias, only variables that are situated far out on the wings of the S were selected, as these are variables that combine both high model influence with high reliability (high covariation and correlation), and contribute to class separation (Figure S4A). However, since the S-plot is susceptible to data matrix changes due to correlation sensitivity and dependency on data structure, the statistical significance and discriminability of these S-plot-derived variables were further investigated using the variable importance in projection (VIP) plot and jackknife confidence intervals (Figure S4B), and finally by evaluation of each variable’s dot plot (not shown). VIP scoring is a metric that summarizes the importance of each variable in driving the observed group or class separation in a classification modelling; and a variable with a VIP score ˃ 1.0 means that the variable contributes more than average to the model, hence its relevance and statistically worth selecting [7, 8, 9]. A dot plot, on the other hand, is similar to a histogram and kernel density estimation (but with algorithmic nuances), computing each observation as a unit: the observations are sorted into “bins” representing variable sub-ranges; and a very strong discriminating variable has no overlap between groups [9, 10]. Finally each of the selected variables was further evaluated using descriptive statistics. Furthermore, the OPLS-DA scores plots were respectively color-coded based on each categorical (descriptive) variable in the models; providing thus a visual distribution of the categorical variables between the two groups. This explorative approach allowed the selection of important descriptive variables, which were further assessed by computing the Chi-Square of independence assessed the significance of the distribution.


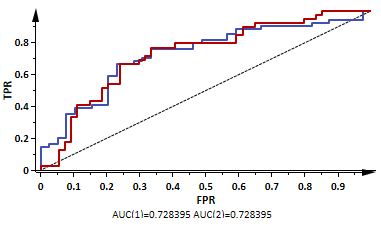


**Figure S3. An example of a typical receiver operator characteristic (ROC) plot:** a ROC plot for the OPLS-DA model (1 + 1 + 0 components, R^2^X = 0.533, Q^2^ = 0.36, CV-ANOVA *P* = 0.001) computed for *VDR* mRNA baseline levels. The ROC plot is a graphical summary of the performance of a binary classifier, plotting the true positive rate (TPR) against the false positive rate (FPR). The sensitivity and specificity of a binary classifier are measured by the area under the ROC curve. An area of 1 represents a perfect classifier. In this case, the AUC = 0.728, which indicates that the computed OPLS-DA model is a good binary classifier.

**
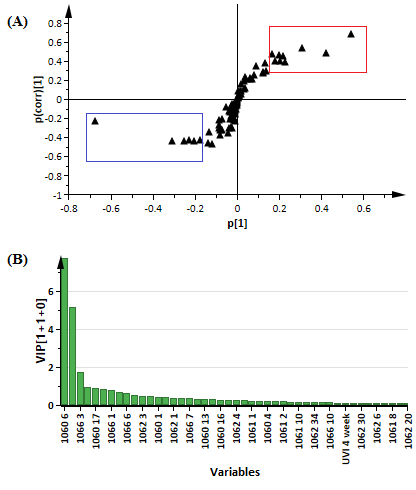
**

**Figure S4. A typical OPLS-DA loading S-plot and Variables Importance for the Projection (VIP) plot for variable selection.** These examples (S and VIP plots) are from OPLS-DA models computed for baseline *VDR*-mRNA and hCAP data, respectively. The loading S-plot (A) aids in the identification of discriminant variables: features combining high model influence (high covariation/magnitude; p1-axis) with high reliability (i.e. smaller risk for spurious correlation; p(corr)1-axis) are statistically relevant discriminating variables. The VIP plot (B) summarizes the importance of continuous variables in the model to explain variation in X and correlation to Y. VIP-values larger than 1 indicate “important” X-variables.

1. **Supplementary results**

**2.1. TLR-VDR pathway genetics differs between races**

To assess whether the identified genetic variation between individuals in the TLR2/1-VDR signaling pathway within the South African population extends to other African and European populations, the 1000 Genomes Deep Catalog of Human Genetic Variation was queried. Similar race-specific genotype frequency distribution between Africans (Yoruba) and Caucasians was observed (Table S2).

**Table S2** **Differential genotype distribution for polymorphic SNPs in the TLR-VDR pathway in the Yoruba (YRI) and Central European Caucasians (CEU) of the 1000 Genomes Project.**

| **Gene** | **Genotype**^a^ | **YRI** | **CEU** | **Pearson's chi-square** | | | |
| --- | --- | --- | --- | --- | --- | --- | --- |
| **Polymorphism**  **(common name)** |  | **Number (%)** | **Number (%)** | ***χ*^2^** | ***df*** | ***P*-value** | **Cramer’s V**^b^ |
| ***GC*** |  |  |  |  |  |  |  |
| rs7041 | GG | 0 (0) | 28 (33) | 79 | 2 | **< 0.001** | 0.678 |
|  | GT | 16 (18) | 43 (51) |  |  |  |  |
|  | TT | 72 (82) | 14 (16) |  |  |  |  |
| rs4588 | AA | 0 (0) | 2 (2) | 32 | 2 | **< 0.001** | 0.430 |
|  | AC | 7 (8) | 37 (44) |  |  |  |  |
|  | CC | 81 (92) | 46 (54) |  |  |  |  |
| ***TLR1*** |  |  |  |  |  |  |  |
| rs5743551 (A7202G) | AA | 81 (92) | 58 (68) | 16 | 2 | **< 0.001** | 0.305 |
|  | AG | 7 (8) | 24 (28) |  |  |  |  |
|  | GG | 0 (0) | 3 (4) |  |  |  |  |
| rs4833095 (N248S) | AA | 2 (2) | 58 (68) | 16 | 2 | **< 0.001** | 0.839 |
|  | AG | 12 (14) | 24 (28) |  |  |  |  |
|  | GG | 74 (84) | 3 (4) |  |  |  |  |
| rs5743618 (I602S) | CC | 0 (0) | 60 (71) | 154 | 1 | **< 0.001** | 0.943 |
|  | CA | 1 (1) | 21 (25) |  |  |  |  |
|  | AA | 87 (99) | 4 (5) |  |  |  |  |
| ***TLR2*** |  |  |  |  |  |  |  |
| rs3804099 (T597C) | CC | 38 (43) | 16 (19) | 13 | 2 | **< 0.010** | 0.276 |
|  | CT | 38 (43) | 46 (54) |  |  |  |  |
|  | TT | 12 (14) | 23 (27) |  |  |  |  |
| ***TIRAP*** |  |  |  |  |  |  |  |
| rs8177374 (S180L) | AA | 0 (0) | 2 (2) | 33 | 2 | **< 0.001** | 0.438 |
|  | AG | 0 (0) | 25 (29) |  |  |  |  |
|  | GG | 88 (100) | 58 (68) |  |  |  |  |
| ***VDR*** |  |  |  |  |  |  |  |
| rs11568820 (Cdx-2) | AA | 86 (98) | 5 (6) | 147 | 2 | **< 0.001** | 0.920 |
|  | AG | 2 (2) | 31 (36) |  |  |  |  |
|  | GG | 0 (0) | 49 (58) |  |  |  |  |
| rs4516035 (GATA) | AA | 87 (99) | 36 (42) | 67 | 2 | **< 0.001** | 0.623 |
|  | AG | 1 (1) | 38 (45) |  |  |  |  |
|  | GG | 0 (0) | 11 (13) |  |  |  |  |
| rs2228570 (*Fok*I^c^) | TT / ff | 2 (2) | 16 (19) | 26 | 2 | **< 0.001** | 0.385 |
|  | TC / Ff | 25 (28) | 40 (47) |  |  |  |  |
|  | CC / FF | 61 (69) | 29 (34) |  |  |  |  |
| rs1544410 (*Bsm*I) | AA / BB | 7 (8) | 23 (27) | 14 | 2 | **< 0.001** | 0.283 |
|  | AG / bB | 38 (43) | 38 (45) |  |  |  |  |
|  | GG / bb | 43 (49) | 24 (28) |  |  |  |  |
| rs7975232 (*Apa*I) | TT / AA | 34 (39) | 34 (40) | 1 | 2 | ns | 0.065 |
|  | TG / Aa | 40 (45) | 34 (40) |  |  |  |  |
|  | GG / aa | 14 (16) | 17 (20) |  |  |  |  |
| rs731236 (*Taq*I) | TT / TT | 45 (51) | 24 (28) | 15 | 2 | **< 0.001** | 0.294 |
|  | TC / Tt | 36 (41) | 38 (45) |  |  |  |  |
|  | CC / tt | 7 (8) | 23 (27) |  |  |  |  |

^a^Alleles used in genotypes represent those of the strand on which the gene is located.

^b^Cramer’s V indicates effect size and varies between 0 and 1: Small 0.07-0.20, medium 0.21-0.34 and large 0.35-1.00.

^c^*Fok*I, *Bsm*I, *Apa*I and *Taq*I genotypes show nucleotides or the presence or absence of restriction sites; lowercase indicating restriction site presence.

**2.2 *VDR* methylation differs between healthy black and white South Africans**

Methylation level differed significantly at several individual CpG sites across the *VDR*. Blacks had significantly higher methylation than Whites at CpG 9 in CGI 1066 (*P* < 0.050, Figure S5A), CpG 8 (*P* < 0.001), 9 (*P* < 0.050), 11 (*P* < 0.050), 12 (*P* < 0.010), 13 (*P* < 0.001), 17 (*P* < 0.050), 19 (*P* < 0.010), 20 (*P* < 0.050), 22 (*P* < 0.001), 25 (*P* < 0.050), 27 (*P* < 0.010), 29-34 (*P* < 0.001), 35 (*P* < 0.050), 40 (*P* <0.050), 44 (*P* < 0.010), 45 (*P* < 0.050), 47 (*P* < 0.050), 49 (*P* < 0.050), 51 (*P* < 0.010), 53 (*P* < 0.050), 54 (*P* < 0.010), and 56 (*P* < 0.010) in CGI 1602 (Figure S5B), and at CpG 3 (*P* < 0.050), 7 (*P* < 0.050) and 9 (*P* < 0.050) in CGI 1061 (*P* < 0.050, Figure S5C). While Blacks had significantly higher methylation than Whites at CpG 1 (*P* < 0.001), 2 (*P* < 0.001), 3 (*P* < 0.001), 4 (*P* < 0.050), and 5 (*P* < 0.001) in CGI 1060, Whites had significantly higher methylation at CpG 8 (*P* < 0.010), 13 (*P* < 0.050), 16 (*P* < 0.001), 17 (*P* < 0.050) and 18 (*P* < 0.010, Figure S5D).

**(A)**

CGI 1066

*

**(B)**

CGI 1062

***

***

v

v

v

v

v

v

v

v

v

v

v

***

***

v

*

**

*

v

v

v

v

v

v

v

v

*

***

**

*

*

**

***

****

**

*

*

*

**

*

*

*

**

**

***

*

v

**(C)**

**(D)**

*

*

*

*

**

***

*

**

***

CGI 1061

*

***

***

***

CGI 1060

**Figure S5. Site-specific *VDR* methylation differed between healthy Black and White South Africans.** The error bar plots show the mean site-specific methylation level for Blacks (black dots, n = 50) and Whites (clear dots, n = 50) at enhancer CGI 1066 (A), primary promoter CGI 1062 (B), gene-body CGI 1061 (C) and 3’-end CGI 1060 (D), quantified by bisulfite pyrosequencing. Pairwise significant differences are shown (**P* < 0.050, ***P* < 0.010, ****P* < 0.001).

**References**

1. Zella LA, Meyer MB, Nerenz RD, Lee SM, Martowicz ML, Pike JW. Multifunctional enhancers regulate mouse and human vitamin D receptor gene transcription. *Mol Endocrinol* 2010; **24**: 128-47.
2. Takai D, Jones PA. Comprehensive analysis of CpG islands in human chromosomes 21 and 22, *Proc Natl Acad Sci USA* 2002; **99**: 3740-5.
3. Meissner A, Mikkelsen TS, Gu H, Wernig M, Hanna J, Sivachenko A et al. Genome-scale DNA methylation maps of pluripotent and differentiated cells. *Nature* 2008; **454**: 766-70.
4. Saccone D, Asani F, Bornman L. Regulation of the vitamin D receptor gene by environment, genetics and epigenetics. *Gene* 2015; **561**: 171-80.
5. Tugizimana F, Piater LA, Dubery IA. Plant metabolomics: a new frontier in phytochemical analysis. *S Afr J Sci* 2013; **34**: 1-8.
6. Wiklund S, Johanson E, Sjöström L, Mellerowicz EJ, Edlund U, Shockcor JP, et al. Visualization of GC/TOF-MS-based metabolomics data for identification of biochemically interesting compounds using OPLS class models. *Anal Chem* 2008; **80**: 115-22.
7. Galindo-Prieto B, Eriksson L, Trygg J. Variable influence on projection (VIP) for OPLS models and its applicability in multivariate time series analysis. *Chemometr Intell Lab* 2015; **146**: 297-304.
8. Wheelock AM, Wheelock CE. Trials and tribulations of ‘omics’ data analysis: assessing quality of SIMCA-based multivariate models using examples from pulmonary medicine. *Mol Biosyst* 2013; **9**: 2589-96.
9. Tugizimana F, Steenkamp PA, Piater LA, Dubery IA. A conversation on data mining strategies in LC-MS untargeted metabolomics: pre-processing and pre-treatment steps. *Metabolites* 2016; **6**: 1-18.

[10]. Wilkinson L. Dot plots. *Am Stat* 1999; **53**: 276-81.
